# Higher order polyploids exhibit enhanced desiccation tolerance in the grass *Microchloa caffra*

**DOI:** 10.1093/jxb/erae126

**Published:** 2024-03-21

**Authors:** Rose A Marks, Paula Delgado, Givemore Munashe Makonya, Keren Cooper, Robert VanBuren, Jill M Farrant

**Affiliations:** Department of Horticulture, Michigan State University, East Lansing, MI 48824, USA; Plant Resilience Institute, Michigan State University, East Lansing, MI 48824, USA; Department of Molecular and Cell Biology, University of Cape Town, Rondebosch 7701, South Africa; Department of Molecular and Cell Biology, University of Cape Town, Rondebosch 7701, South Africa; Department of Molecular and Cell Biology, University of Cape Town, Rondebosch 7701, South Africa; Washington State University, Irrigated Agriculture Research and Extension Centre, Prosser, WA 99350, USA; Department of Molecular and Cell Biology, University of Cape Town, Rondebosch 7701, South Africa; Department of Horticulture, Michigan State University, East Lansing, MI 48824, USA; Plant Resilience Institute, Michigan State University, East Lansing, MI 48824, USA; Department of Molecular and Cell Biology, University of Cape Town, Rondebosch 7701, South Africa; Hong Kong Baptist University

**Keywords:** Desiccation tolerance, local adaptation, natural variation, polyploidy, resilience, resurrection plants

## Abstract

Desiccation tolerance evolved recurrently across diverse plant lineages to enable survival in water-limited conditions. Many resurrection plants are polyploid, and several groups have hypothesized that polyploidy contributed to the evolution of desiccation tolerance. However, due to the vast phylogenetic distance between resurrection plant lineages, the rarity of desiccation tolerance, and the prevalence of polyploidy in plants, this hypothesis has been difficult to test. Here, we surveyed natural variation in morphological, reproductive, and desiccation tolerance traits across several cytotypes of a single species to test for links between polyploidy and increased resilience. We sampled multiple natural populations of the resurrection grass *Microchloa caffra* across an environmental gradient ranging from mesic to xeric in South Africa. We describe two distinct ecotypes of *M. caffra* that occupy different extremes of the environmental gradient and exhibit consistent differences in ploidy, morphological, reproductive, and desiccation tolerance traits in both field and common growth conditions. Interestingly, plants with more polyploid genomes exhibited consistently higher recovery from desiccation, were less reproductive, and were larger than plants with smaller genomes and lower ploidy. These data indicate that selective pressures in increasingly xeric sites may play a role in maintaining and increasing desiccation tolerance and are mediated by changes in ploidy.

## Teaser

We describe two diverging ecotypes of the resurrection grass *Microchloa caffra* occuring along an environmental aridity gradient . Our findings provide intriguing evidence that desiccation tolerance may be mediated by ploidy changes.

## Introduction

Desiccation-tolerant organisms can withstand nearly complete drying of their tissues without dying (Bewley, 1979; Bewley and Krochko, 1982; Oliver *et al.*, 2020). Plants with this ability in their vegetative tissues are commonly referred to as resurrection plants because of their dramatic ability to revive from a water potential of less than –100 MPa. Desiccation tolerance evolved in plants >500 million years ago and played a critical role in enabling the transition from aquatic to terrestrial environments by early land plants (Oliver *et al.*, 2000). Throughout evolutionary time, desiccation tolerance was lost (or suppressed) in the vegetative tissues of most vascular plants, but was maintained in many early diverging lineages (e.g. bryophytes, ferns, and fern allies) and some specialized tissues (e.g. seeds, spores, and pollen) (Proctor and Tuba, 2002; Oliver *et al.*, 2005). Desiccation tolerance then re-evolved convergently in the vegetative tissues of diverse angiosperms, probably through rewiring of ancestral pathways maintained in seeds and other specialized tissues (Costa *et al.*, 2017; VanBuren, 2017; VanBuren *et al.*, 2017). As a result, extant resurrection plants are phylogenetically and morphologically diverse, with representatives in every major land plant lineage except gymnosperms. Resurrection plants are found on all seven continents (Alpert, 2006) where they often occur in closely intertwined communities on isolated rock outcrops (Porembski, 2011) and other extremely water-limited habitats (Marks *et al.*, 2021). Resurrection plants have received growing research attention over the past two decades (Tebele *et al.*, 2021) due to their astonishing resilience, but many questions remain regarding the evolutionary history, genetic mechanisms of desiccation tolerance, and natural diversity within and across species of resurrection plants (Marks *et al.*, 2021).

Plant evolution has been continually shaped by genome duplication, and an estimated 70% of flowering plants have experienced at least one whole-genome duplication event in their evolutionary history (Soltis *et al.*, 2014). This has resulted in numerous polyploid lineages that possess anywhere from three to >1000 homologous chromosomes (Stuessy and Weiss-Schneeweiss, 2019). Among other effects, the resulting chromosomal redundancy may function to enhance adaptability to extreme environmental conditions, as the duplicated genes offer a broader genetic basis for the evolution of potential stress tolerance mechanisms (Comai, 2005). Polyploidy results in functional redundancy as well as adaptive conflicts that can lead to subfunctionalization, neofunctionalization, or silencing of duplicated genes, and foster the evolution of novel traits which, in turn, can lead to the development of new and emergent phenotypes (Wendel, 2000). As a result, polyploidy plays a crucial role in driving plant diversification, speciation, and adaptation to varied ecological niches (Otto, 2007; Te Beest *et al.*, 2012; Soltis *et al.*, 2015).

Many resurrection plants are polyploid, and several groups have hypothesized that polyploidy contributed to the evolution of desiccation tolerance (Giarola *et al.*, 2017), speculating that genome duplication could allow desiccation tolerance mechanisms maintained in seeds to be uniquely regulated in vegetative tissues through the neofunctionalization of regulatory networks. However, testing this hypothesis is challenging because of the vast phylogenetic distance between resurrection plants, the rarity of tolerant lineages (Oliver *et al.*, 2020; Marks *et al.*, 2021), the prevalence of polyploidy across plants (Otto and Whitton, 2000; Husband *et al.*, 2013; Soltis *et al.*, 2015; Van de Peer *et al.*, 2021), and the association of polyploidy with numerous other adaptive traits (Wei *et al.*, 2019; Van de Peer *et al.*, 2021). A natural system of variable ploidies within a single species would minimize confounding factors and could be used to test for associations between polyploidy and desiccation tolerance.

The diverse and climate-resilient *Chloridoideae* subfamily of *Poaceae* has >40 desiccation-tolerant species (Marks *et al.*, 2021), and an estimated 90% of species within this clade are polyploid (Davidse *et al.*, 1986; Roodt and Spies, 2003). Despite the prevalence of polyploidy within this lineage, some desiccation-tolerant Chloridoid grasses such as *Oropetium thomaeum*, *O. capense*, *Tripogon minimus*, *T. spicatus*, and *T. loliiformis* have very small diploid genomes of ~220–270 Mb (Mehra and Sharma, 1975; Bartels and Mattar, 2002; VanBuren *et al.*, 2015). In contrast, other desiccation-tolerant Chloridoid grasses including *Eragrostis nindensis* and *Sporobolus stapfianus* are tetraploid (Pardo *et al.*, 2020; Chávez Montes *et al.*, 2022) so there is no clear association between polyploidy and desiccation tolerance within the Chloridoid grasses. These patterns indicate that polyploidy is not required for desiccation tolerance, but whether polyploidy can enhance tolerance within a single species has not been tested. Most studies on desiccation tolerance have focused on a single accession or ecotype (Marks *et al.*, 2021), few studies have surveyed natural variation in desiccation tolerance across populations of a single species (but see Marks *et al.*, 2019a, 2022), and the relationship between ploidy and desiccation tolerance within a single species has not been explored.

Here, we surveyed natural variation in ploidy, desiccation tolerance, and morphological and reproductive traits in a single Chloridoid grass species. We sampled multiple populations and accessions of the resurrection grass *Microchloa caffra* across an environmental gradient spanning ~500 km in the Mpumalanga and Limpopo provinces of northeastern South Africa. We employed both field and common garden studies to distinguish between plastic and genetic differences in phenotypes. We tested the hypothesis that persistent differences in desiccation tolerance, morphology, and reproduction exist along environmental gradients, and that phenotypic differences are modulated by changes in genome size and ploidy. We predicted that desiccation tolerance would parallel the environmental gradient, with plants from dryer habitats exhibiting increased recovery from desiccation compared with plants from wetter habitats, and that variation in desiccation tolerance would be positively associated with genome size, pointing towards a role for elevated ploidy in enhancing desiccation tolerance. We also tested for trait associations between desiccation tolerance and other functional traits that could point towards relevant trade-offs and be used to guide applied objectives. Our findings provide insight into how desiccation tolerance evolves on relatively short time scales and highlight the complex interplay between stress tolerance, ploidy, and other life history traits.

## Materials and methods

### Study species

**Fig. 1.**
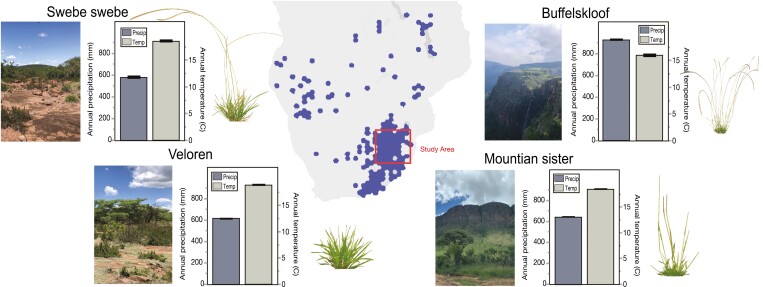
Distribution of *Microchloa caffra* taken from GBIF.org (22 August 2023) GBIF Occurrence Download https://doi.org/10.15468/dl.2stdgx. The study area spans ~500 km in Mpumalanga and Limpopo provinces of northeastern South Africa. Representative images of the four sampling regions and plants are included, and bioclimatic data on annual precipitation and temperature are plotted from WorldClim (Fick and Hijmans, 2017). Multiple populations were sampled within each region.

*Microchloa caffra* Nees is a perennial grass in the Chloridoid subfamily of *Poaceae* commonly known as pincushion grass. It is distributed across Southern Africa from Uganda to South Africa, with the highest density of plants in northeastern South Africa (Fig. 1). Plants occur in seasonally dry areas with summer rainfall in shallow soils on rocky outcrops or inselbergs, locally referred to as ruwari. The vegetative tissues of *M. caffra* are desiccation tolerant and can survive repeated cycles of de- and rehydration within a single growing season.

### Field collections and phenotyping

Live specimens and seeds of *M. caffra* were collected from 11 populations in four major regions across Mpumalanga and Limpopo provinces of South Africa from December 2021 to January 2022 (Fig. 1) and a voucher specimen was deposited in the National Herbarium Pretoria (specimen number PRE1004810-0). The GPS coordinates and elevation of each population were logged with a Garmin 64csx GPS, and bioclimatic data on annual precipitation and temperature were extracted from WorldClim (Fick and Hijmans, 2017) based on the GPS coordinates of each site using the R package raster (Supplementary Table S1).

Seven plants from each population (*n*=77) were phenotyped in the field, three of which (*n*=33) were collected, potted, and brought into cultivation for seed bulking and further experimentation. In the field, we measured the length and width of three randomly selected leaves and panicles from each plant and counted the total number of panicles on each plant. Measurements were taken to the nearest 0.5 mm.

### Seed bulking and germination tests

Seeds were collected from each of the 33 accessions (3 plants from 11 populations) and transported to Michigan State University under United States Department of Agriculture (USDA) permit #537-22-37-10071 and according to the specifications in a Material Transfer Agreement established between Drs Jill M. Farrant, Robert VanBuren, and Rose A. Marks. Seeds were cold stratified for 2 weeks at 4 °C and then germinated on a standard propagation mix of 50:50 redi-earth and suremix under controlled conditions in growth chambers set to ~400 µmol m^–2^ s^–1^ light with a 16 h photoperiod and 28/18 °C day/night temperature. Depending on availability, five or 10 seeds were sown for each of the 33 accessions. Germination percentage was recorded at 4 weeks and seedlings were then transplanted into individual pots for experimental treatments. Replicates of each accession were split into two sets, one for morphological phenotyping (*n*=102) and the other for desiccation treatment (*n*=71).

### Phenotyping seed-grown plants

Plants selected for phenotyping were photographed every 2 weeks for 12 weeks (beginning at 6 weeks old and extending to 18 weeks old) using a Nikon D5600 DSLR Camera with a 35 mm lens to monitor growth. Profile and overhead images were taken with scale and color standards included in each photograph. At 11 weeks old, plants were manually measured to quantify leaf and panicle length and width—paralleling field measurements. The number of panicles on each plant was manually counted when plants were 8 months old, and plants were maintained for seed production. The time to panicle emergence and seed maturity was monitored. The first panicles emerged when plants were between 6 and 8 weeks old, and the entire cycle from seed to seed was completed within ~6–10 months, depending on the accession.

### Genome size and ploidy estimations

Ploidy was estimated for a single representative *M. caffra* accession from Buffelskloof using a K-mer-based approach (Ranallo-Benavidez *et al.*, 2020). We selected the accession with the smallest haploid genome size (~1 Gb) based on flow cytometry results. To generate sequence data, healthy green tissue was harvested and flash-frozen in liquid nitrogen. Tissue was ground by hand using a mortar and pestle for >20 min to achieve maximal grinding. Genomic DNA was extracted by isolating nuclei with the Circulomics Nuclei Isolation kit and then extracting high molecular weight DNA using the Circulomics Nanobind Plant Nuclei Big DNA kit according to the manufacturer’s instructions. Genomic DNA was sequenced at the University of Georgia Sequencing Core to generate ~34 Gb of PacBio high fidelity (HiFi) sequence data. K-mers were counted from the high-quality circularized consensus reads using Jellyfish (V2.3.0) (Marçais and Kingsford, 2011) with default parameters and a K-mer length of 21 bp. A histogram of the K-mer distribution was used as input for GenomeScope2.0, and the monoploid genome size, ploidy, and likely origin of polyploid events (allo or auto) were calculated. GenomeScope2.0 uses Bayesian inference to estimate the heterozygosity of the genome and test the hypothesis of autopolyploidy (aaab) versus allopolyploidy (aabb).

The genome size of each accession was estimated by flow cytometry at Plantploidy.com to generate 2C DNA values. Briefly, healthy leaf tissue was harvested from each accession and nuclei were isolated and stained according to standard protocols. The stained nuclei were then run on a BD Accuri™ C6 Plus Flow Cytometer. Fluorescence was measured and doublets were removed. *Hosta plantaginea* and hybrid daylily *Hemerocallis* ‘Purple Pixie Gumdrop’ were used as internal references and 2C DNA values were calculated (see Appendix S1). The estimated genome sizes were compared with the genome size of the hexaploid reference accession to estimate the ploidy of each accession.

### Screening desiccation tolerance phenotypes in field-collected plants

Three *M. caffra* plants from each of the 11 populations (*n*=33) were collected from the field, potted, and transported to the University of Cape Town where they were maintained in a climate-controlled greenhouse for 1 month. Selected accessions from the two most environmentally distinct sites were targeted for desiccation assays. Six individual plants from Swebe Swebe and five from Buffelskloof were transferred to a growth room maintained at 20 °C with a 16 h photoperiod. After 1 week, they were subjected to a desiccation treatment and their ability to recover was assessed. Plants were watered to field capacity (full soil saturation), after which water was withheld and the plants dehydrated naturally over the following 9 d. Plants were then rehydrated by rewatering with dH_2_O and their recovery was assessed.

Plants were sampled every 48 h during the drying process and again 48 h after rehydration. At each sampling time point, we measured maximum quantum yield of PSII (*F*_v_*/F*_m_) and relative water content (RWC), and took TEM images of leaf tissue. All measurements were taken in triplicate, with three leaves sampled per plant for each variable. Briefly, *F*_v_*/F*_m_ was measured on dark-acclimated leaves using a PAR-Fluorpen FP 110/D at 455 nm to quantify PSII efficiency as an indicator of physiological stress. RWC was quantified to confirm the water status of tissues by measuring leaf mass immediately after collection (fresh mass), after 48 h submerged in water in darkness at 4 °C (turgid mass), and again after 48 h in a 70 °C drying oven (dry mass). RWC was calculated as (fresh mass–dry mass)/(turgid mass–dry mass). Leaves sampled for TEM were fixed and processed following Cooper and Farrant (2002) to visualize subcellular changes during desiccation. Briefly, leaves were fixed overnight at 4 °C in 2.5% glutaraldehyde in 0.1 M phosphate buffer (pH 7.4) containing 0.5% caffeine, post-fixed by washing in 1% osmium phosphate buffer, and then dehydrated in graded ethanol (30, 50, 70, 85, 95, and 100%) and washed twice in 100% acetone. The samples were then incubated overnight at 4 °C in a 1:1 solution of acetone:Spurr’s resin, the concentration of which was gradually increased over the course of 4 d until 100% Spurs resin was reached. The resulting samples were placed into molds and allowed to harden at 60 °C for 24 h. Fixed tissues were sectioned using a Richart Ultracut S Ultramicrotome and mounted on copper grids. Sections were stained with uranyl acetate and lead citrate for contrast and visualized on an FEI Tecnai T20 transmission electron microscope. A total of 238 images were taken and systematically assessed.

### Screening desiccation tolerance phenotypes in seed-grown plants

Comprehensive desiccation assays were performed on a replicated set of all 33 accessions grown from seed under common conditions. Progeny from each of the 33 accessions (*n*=71) were subjected to a desiccation treatment at Michigan State University that was similar to, but more comprehensive than, the treatment imposed on field-collected plants at University of Cape Town. Six-week-old plants were watered to field capacity, after which water was withheld and plants were allowed to desiccate naturally over the course of 4 weeks. Plants were sampled once a week on Tuesdays at 14.00 h. After 4 weeks, plants were rehydrated with dH_2_O and sampled 24, 48, 96, and 192 h after rehydration. At each sampling time point, overhead and profile photographs were taken and *F*_v_*/F*_m_ was quantified on dark-acclimated leaves using a Opti-Sciences OS30p+ chlorophyll fluorometer with the default test parameters. RWC was measured immediately before rehydration (as described above) to confirm that plants had reached a desiccated state.

Overhead photographs from the final recovery time point (192 h post-rehydration) were analyzed using ImageJ to quantify the percentage of tissue that survived and recovered from desiccation. Images were segmented in ImageJ following the protocol described at Pride *et al.* (2020). Briefly, plant canopies were separated from the background using the color thresholding tool with hue set to 1–170, saturation set to 69–255, and brightness set to 50–255. The background was cleared and outliers removed. Senesced tissue was isolated from healthy tissue by adjusting the thresholding parameters to hue of 1–35, saturation of 0–255, and brightness of 0–255. Healthy recovered tissue was isolated by setting the thresholding parameters to hue of 35–255, saturation of 0–255, and brightness of 0–255. The areas of the entire canopy, the senesced tissue, and the recovered tissue were computed, and percent recovery was calculated as the proportion of recovered tissue relative to the entire canopy for each replicate.

### Statistical analyses

All statistical analyses were run in JMP 15 (SAS Institute Inc., Cary, NC, USA). We tested for differences across populations and regions in the response variables of leaf length, panicle length, panicle number, percent recovery from desiccation, and genome size using generalized mixed linear models. Plant ID was included in each model as a random effect, and population was nested within region. We tested for differences in morphological traits (leaf length, panicle length, panicle number) separately for field-collected plants (parental generation) and plants cultivated in common conditions (progeny generation). We computed the *R*^2^ values across field and common garden plants by running separate mixed effects linear models to test the effect of parental phenotypes on progeny phenotypes, with parental plant ID included in the model as a random effect. Desiccation responses were also quantified separately for field-collected and common garden plants because different response variables were measured in the two desiccation assays. For field-collected plants, we tested for differences in *F*_v_*/F*_m_ and RWC with generalized linear models that included the fixed effects of region, time point, and the interaction between the two. For plants cultivated under common conditions, we tested for differences in *F*_v_*/F*_m_ and percent recovery. We tracked *F*_v_*/F*_m_ throughout the entire time course and tested for differences across regions using repeated measures ANOVA. For percent recovery, we used a mixed effects linear model to test the effect of population and region on recovery at 192 h post-rehydration, with plant ID included as a random effect.

Dimension reduction via principal component analyses (PCAs) was used to visualize emergent patterns in this multivariate dataset. Trait associations and correlations were computed for mean leaf length, panicle length, panicle number, genome size, and percent recovery for each accession, and the site characteristics of elevation, annual precipitation, and annual temperature using Pearson’s correlation. A correlation matrix was generated by restricted maximum likelihood (REML) across all of these factors, and singular value decomposition (SVD) was used to obtain eigenvectors and eigenvalues for PCA.

## Results

### Field collections and study sites

We collected 33 accessions of *M. caffra* across 11 populations in four major areas of Mpumalanga and Limpopo provinces in South Africa (Supplementary Dataset S1). Study sites range from low elevation xeric to high elevation mesic areas and span ~500 km in distance, 720 m in elevation, ~400 mm in mean annual rainfall, and 3 °C in mean annual temperature (Fig. 1; Supplementary Table S1) (Marks *et al.*, 2022). In general, sites within Buffelskloof are the wettest, coolest, and highest elevation. Sites within Veloren and Sister Mountains are intermediate, and sites within Swebe Swebe are the hottest, driest, and lowest elevation.

### Morphological and reproductive phenotypes parallel environmental gradients

**Fig. 2.**
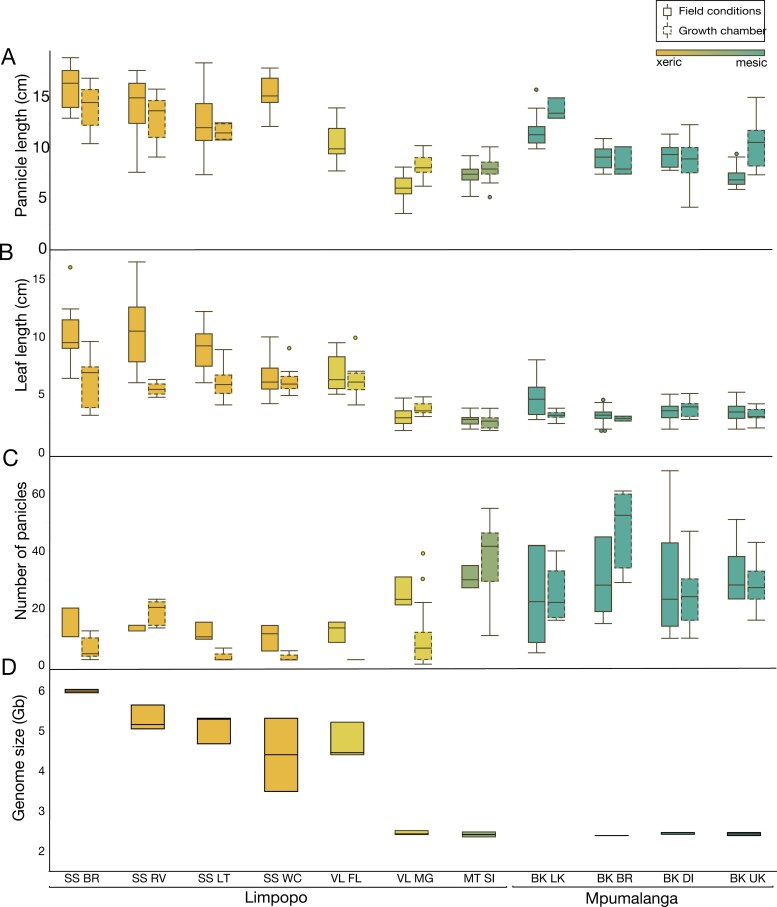
Morphological and reproductive phenotypes of plants in field and common garden conditions. Regions are ordered from xeric to mesic, and sites are ordered from low to high elevation within each region. (A) Mean panicle number, (B) mean panicle length, and (C) mean leaf length are shown for both field and common garden plants. (D) Genome size variation across the study area. SS=Swebe Swebe, VL=Veloren, MT SI=Mountain sister, and BK=Buffelskloof. Secondary letters on the *x*-axis indicate sites within the region: BR=brown river, RV=river view, LT=lazy tree, WC=wasp corner, FL=felicite, MG=main gate, SI=sister, LK=low kloof, BR=breakfast rock, DI=drop in, UK=upper kloof.

Significant morphological and reproductive differences across study sites were identified in field and common garden specimens. Plants were sampled along an environmental gradient and, for field-collected plants, those from the higher elevation cooler mesic areas had shorter leaves (*P*<0.0001) and panicles (*P*<0.0001) relative to plants from lower elevation hotter xeric areas which had longer leaves and panicles (Fig. 2). Interestingly, the plants from higher elevation cool mesic sites produced more panicles than the plants from lower elevation hot xeric sites (*P*=0.0012).

Phenotypic differences were persistent when plants were cultivated under common conditions. Paralleling field phenotypes, plants from the high elevation mesic sites were consistently smaller with more panicles than plants from lower elevation xeric sites which were larger, with longer leaves (*P*<0.0001) and panicles (*P*<0.0001), but fewer overall panicles (*P*<0.0001) (Fig. 2A–C). While the most distinct differences in phenotypes were observed across regions, there are also noteworthy differences across sites within regions. Phenotypic differences between sites within regions also parallel environmental gradients of elevation and temperature. Correlations between parental and progeny phenotypes were tight, suggesting a high degree of stability in morphological traits. *R*^2^ values were 0.837 for leaf length, 0.662 for panicle length, and 0.859 for panicle number. However, phenotypic differences were more variable and pronounced in field-collected specimens relative to plants cultivated in common conditions, suggesting an additive effect of genotypic and plastic differences.

We estimated the ploidy of *M. caffra* and tested if differences in genome size existed across populations. Initially, we quantified the ploidy of a single representative accession from Buffelskloof using whole-genome sequencing data and a K-mer approach. The K-mer plot shows six distinct peaks, indicating that this accession is hexaploid (2*n*=6×), with a monoploid genome size of ~300–400 Mb and heterozygosity of 8.17% (Supplementary Fig. S1; Marks *et al.*, 2023), similar to other Chloridoid grasses, which tend to have more compact genomes than most grass lineages (VanBuren *et al.*, 2015, 2018, 2020; Pardo *et al.*, 2020; Chávez Montes *et al.*, 2022). The distribution of K-mers in the *M. caffra* genome and the prevalence of ‘aaab’ over ‘aabb’ alleles is consistent with autopolyploidy and high genetic diversity (Ranallo-Benavidez *et al.*, 2020).

We then used flow cytometry to estimate genome size of each of the 33 *M. caffra* accessions included in the current study (see Appendix S1). We observed substantial variation in genome sizes across populations and regions (Fig. 2D). Genome sizes varied by >2-fold from 2.35 Gb to 6.04 Gb per 2C, and the plants from lowland xeric sites have consistently and significantly larger genome sizes than the plants from the high elevation mesic sites (*P*<0.0001). Using the hexaploid reference accession as a baseline, we estimate that ploidy varies from hexaploid (2*n*=6×) in the high elevation sites to dodecaploid (2*n*=12×) or more in the low elevation sites. Within the xeric low elevation sites, genome size ranges from 3.5 Gb to 6 Gb per 2C, so intermediate and higher order ploidies above dodecaploid are certainly possible, but need to be confirmed via karyotyping.

### Higher order polyploids exhibit enhanced recovery from desiccation

Comprehensive desiccation assays were performed on a replicated set of all accessions grown from seed under common conditions. The RWC of all samples was confirmed at <10% prior to rehydration, indicating that all plants were fully desiccated prior to rehydration.

**Fig. 3.**
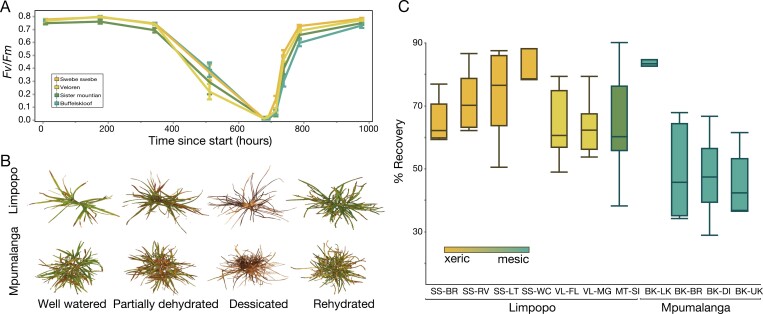
Plant responses to dehydration treatment. (A) Images of representative plants throughout the desiccation and rehydration time course. (B) Photochemical efficiency of PSII (*F*_v_*/F*_m_) of plants during a controlled drying time course. (C) The proportion of tissue that recovered after desiccation was variable across populations. Plants from the most xeric study sites recovered more completely from desiccation compared with plants from mesic sites. Regions are ordered from xeric to mesic and sites are ordered from low to high elevation within each region. SS=Swebe Swebe, VL=Veloren, MT SI=Mountain sister, and BK=Buffelskloof. Secondary letters on the *x*-axis indicate sites within the region: BR=brown river, RV=river view, LT=lazy tree, WC=wasp corner, FL=felicite, MG=main gate, SI=sister, LK=low kloof, BR=breakfast rock, DI=drop in, UK=upper kloof.

*F*_v_ */F*_m_ declined at similar rates and recovered to near baseline levels in all plants from all sites (Fig. 3B) during the desiccation–rehydration time course. *F*_v_*/F*_m_ remained near baseline in all plants for the first 2 weeks of water withholding and then declined steadily over the following 2 weeks. Under desiccated conditions, *F*_v_*/F*_m_ reached, or approached, zero in all accessions, but recovered rapidly during rehydration, returning to near baseline levels after only 4 d. One week after rewatering, tissues were fully functional in all accessions. Subtle differences in *F*_v_*/F*_m_ at intermediate time points were insignificant but may translate into more substantial differences in gas exchange and carbon assimilation (Seaton and Walker, 1990), and future studies should include more detailed physiological assessments of drying dynamics.

Despite similar rates of drying and rehydrating, we detected significant differences across sites (*P*=0.0002) and regions (*P*=0.0004) in the proportion of tissue that recovered from desiccation (Fig. 3C). In general, plants from the higher elevation mesic regions had lower percent recovery than plants from the lower elevation xeric sites (Fig. 3A). Differences in recovery were also evident across sites within regions and were associated with environmental differences in elevation and temperature.

For field-collected plants, the *F*_v_*/F*_m_ and leaf RWC dropped to a lower absolute level in plants from the lowland xeric sites (Swebe Swebe and Limpopo) relative to plants from the higher elevation mesic sites (Buffelskloof and Mpumalanga) (Supplementary Fig. S2). However, the lowland plants recovered more rapidly than plants from the high elevation mesic sites, represented by the significant interaction term of region and time point for both *F*_v_*/F*_m_ (*P*<0.0001) and RWC (*P*=0.0483) (Supplementary Fig. S2). The differences in rate and extent of drying in the two populations could be due to differences in phenological (e.g. leaf area), biochemical (e.g. osmotic potential), or soil properties.

**Fig. 4.**
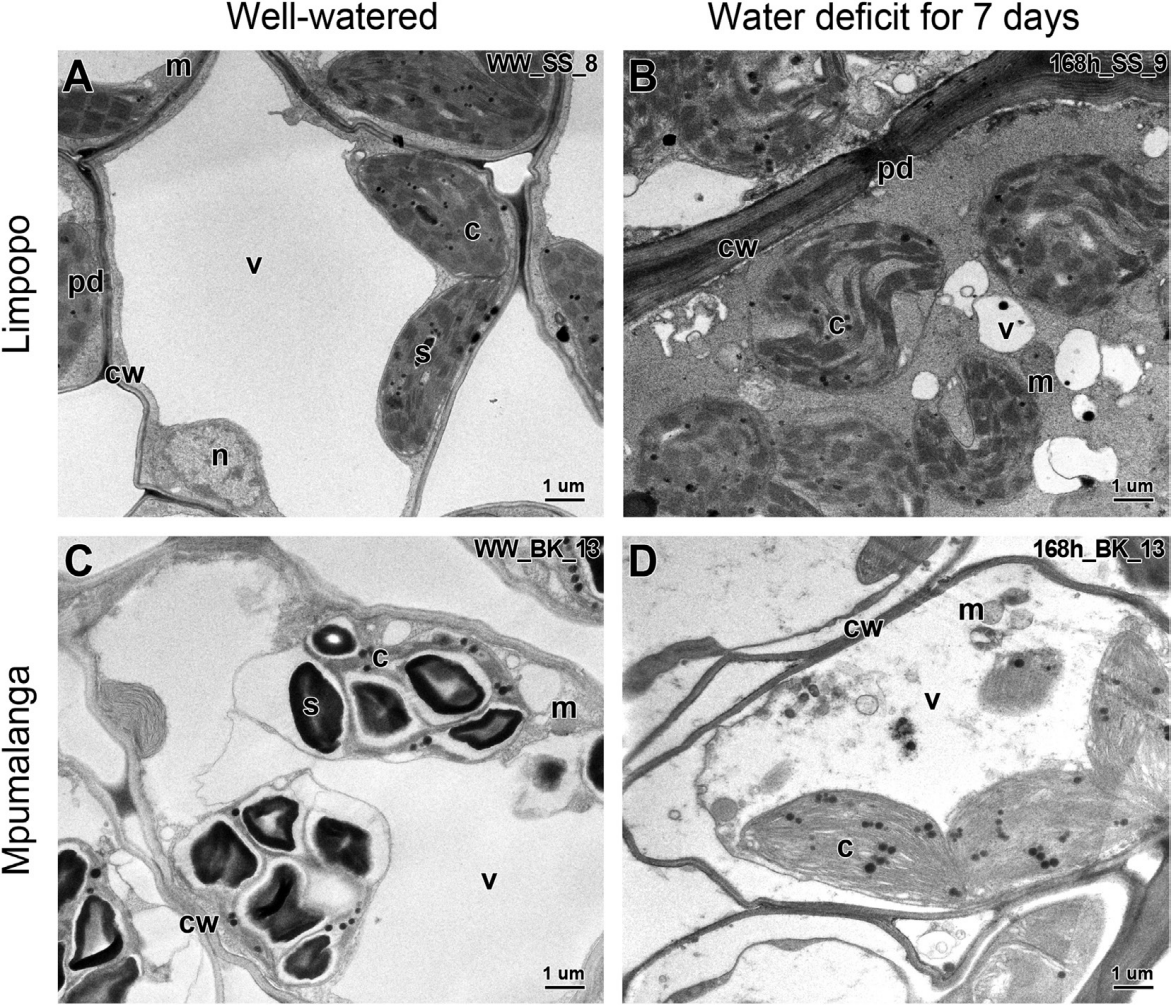
Transmission electron micrographs of mesophyll cells. Plants were collected from Mpumalanga (C and D) and Limpopo (A and B) imaged under partial water deficit (A and C) and substantial water deficit (B and D). Abbreviations: CW=cell wall; C=chloroplast; M=mitochondrion; PD=plasmodesmata; S=starch; V=vacuole.

Subcellular morphology showed characteristic signs of anhydrobiosis in both Buffelskloof (Mpumalanga) and Swebe Swebe (Limpopo) plants. Plants from Swebe Swebe reached a desiccated state after 7 d of dehydration, whereas those from Buffelskloof had reached only ~30% RWC at this stage (Supplementary Fig. S2). In the hydrated state, mesophyll cells of the two populations were typical of metabolically active tissues, with large vacuoles, and mitochondria and chloroplasts with well-defined membranes (Fig. 4A, C; Supplementary Fig. S3A, D, G, J). After 7 d of dehydration, the subcellular organization of the plants from Swebe Swebe was well preserved, with little evidence of plasmalemma withdrawal, well-defined plasmodesmata, intact organelles, densely stained cytoplasm, and numerous small vacuoles (Fig. 4B; Supplementary Fig. S3C, F), typical of the desiccated state reported for other resurrection plants (Farrant *et al.*, 2017). The loss of starch grains during drying indicates mobilization of these reserves for energy. Although the plants from Buffelskloof did not reach an air-dry state in this experiment, it is clear from ultrastructural studies that tissues are likely to be viable, but yet undergo complete re-organization and stabilization of cytoplasm that occurs in resurrection plants during the loss of this final ~30% of water. Cellular membranes and organelles were intact, chloroplastic starch storage had been metabolized, and some subdivision of vacuoles had begun (Fig. 4D; Supplementary Fig. S3I, L). While there is some evidence of plasmalemma withdrawal in drying tissues from Buffelskloof, this is likely to be due to the aqueous fixation protocol used. Mitochondria appear in a highly active state (Fig. 4D; Supplementary Fig. S3I, L), typical of entering the final stages of desiccation in resurrection plants (Farrant *et al*, 2017).

### Functional syndromes delineating the mesic and xeric *M. caffra* ecotypes

**Fig. 5.**
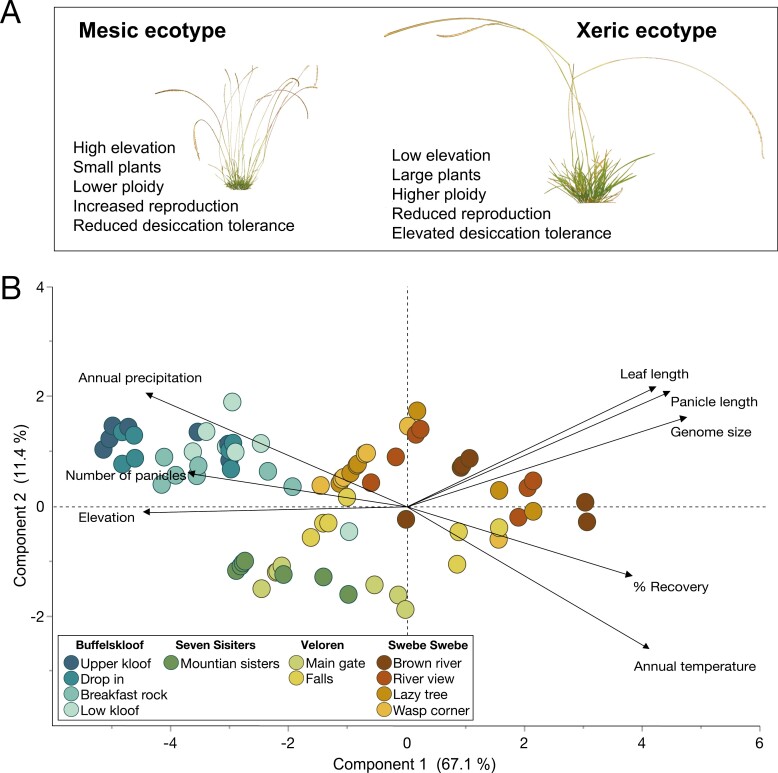
Summary of ecotype differences across the study area. (A) Major features delineating the two ecotypes. (B) Principal component analysis (PCA) of leaf length, panicle length, panicle number, recovery from desiccation, genome size, elevation, annual precipitation, and annual temperature. Samples are colored by site.

We identified numerous positively and negatively correlated traits that, when taken together, describe two diverging ecotypes of *M. caffra*. Plants from high elevation mesic sites had smaller leaves and panicles, were highly reproductive, reduced desiccation tolerance, and lower ploidy. In contrast, plants from the xeric lowland sites had longer leaves and panicles, were less reproductive, more desiccation tolerant, and had higher ploidy (Fig. 5A). Leaf length, panicle length, and genome size were positively correlated. Percent recovery was positively associated with genome size (*R*=0.52), panicle length (*R*=0.58), and leaf length (*R*=0.46), but negatively associated with number of panicles (*R*= –0.54) and elevation (*R*= –0.57). Elevation was positively associated with the number of panicles (*R*=0.54), but negatively correlated with genome size (*R*= –0.74), panicle length (*R*= –0.68), and leaf length (*R*= –0.70) (Table 1). All reported correlations were statistically significant with *P*-values <0.01. PCA explained a considerable amount of the variation in the dataset, with PC1 accounting for 67.1% and separating samples along a gradient of elevation, moisture, genome size, and desiccation tolerance. PC2 explains another 11.4% of the variability in these data and separated samples on more subtle differences in morphology (Fig. 5B). These findings point towards the existence of two ecotypes of *M. caffra* that occur along an environmental gradient in water availability and elevation.

**Table 1.** Environmental and trait correlations

	Annual precipitation	Annual temperature	Elevation	Recovery	Genome size	Leaf length	Panicle length	No. of panicles
Annual precipitation	1.00							
Annual temperature	–0.90	1.00						
Elevation	0.63	–0.70	1.00					
Recovery	–0.61	0.57	–0.57	1.00				
Genome size	–0.70	0.58	–0.74	0.52	1.00			
Leaf length	–0.61	0.48	–0.70	0.46	0.92	1.00		
Panicle length	–0.52	0.46	-0.68	0.58	0.83	0.77	1.00	
No. of panicles	0.58	–0.45	0.54	–0.54	–0.60	–0.58	–0.39	1.00

The correlations were generated by restricted maximum likelihood (REML) and used for principal component analysis (PCA). Positive correlations are shown in red and negative correlations are shown in blue. All correlations were significant with *P*-values <0.01.

## Discussion

Polyploidy has enabled the evolution of complex and emergent traits (Huminiecki and Conant, 2012), including many adaptations related to resilience in plants (Otto, 2007;  Te Beest *et al.*, 2012; Soltis *et al.*, 2015; Van de Peer *et al.*, 2021). Some groups have hypothesized that polyploidy could enhance the evolution of desiccation tolerance traits (Giarola *et al.*, 2017) through either the neo-functionalization of duplicated genes to acquire novel functions (Lynch and Conery, 2000) that may enhance desiccation tolerance, or an increase in gene dosage (Adams and Wendel, 2005), leading to higher abundance of enzymes, structural proteins, and critical end-point metabolites that play important roles in desiccation tolerance. Here, we used a plant system exhibiting natural variation along an environmental gradient to test for associations between polyploidy and desiccation tolerance. We identified persistent variation in recovery from desiccation in the resurrection grass *M. caffra* (Fig. 3) that is associated with differences in ploidy, environmental conditions, and plant morphology (Fig. 5). Accessions with higher ploidy were found at drier sites and displayed greater desiccation tolerance compared with their lower ploidy counterparts.

The role of polyploidy in the evolution of plant desiccation tolerance is an open question, and although we provide evidence that polyploidy enhances tolerance in *M. caffra*, there are important confounding factors to consider. Most Chloridoid grasses (~90%) are polyploid (Davidse *et al.*, 1986; Roodt and Spies, 2003), making it difficult to distinguish causal and correlative relationships when placing ploidy within the context of emergent traits. Desiccation-tolerant Chloridoid grasses within *Oropetium* and *Tripogon* are generally diploid with small, compact genomes, suggesting that polyploidy is not necessary for evolving desiccation tolerance within this clade. More broadly, natural polyploids arise mostly through unreduced gametes (Soltis and Soltis, 2009), and environmental stresses increase the rate of these errors during meiosis (Mason *et al.*, 2011; Pecrix *et al.*, 2011; De Storme *et al.*, 2012; Van de Peer *et al.*, 2021). The extreme environments that resurrection plants inhabit and the repeated cycles of drying may increase the rate of polyploid formation within these species (Gutschick and BassiriRad, 2003). Polyploidy has been implicated in local adaptation in other species and functional traits (Baker *et al.*, 2017; McIntyre and Strauss, 2017; Wei *et al.*, 2019; López-Jurado *et al.*, 2022), and our data suggest that it may play a role in enhancing desiccation tolerance in specific ecological contexts. Rather than enabling desiccation tolerance, we suggest a more nuanced role of polyploidy, where genome duplication improves desiccation tolerance traits by providing more raw genetic material for possible sub- and neo-functionalization. Polyploidy could enable gene networks previously restricted to specific regulatory contexts (e.g. seeds) to be co-opted by other developmental contexts and tissues (e.g. vegetative tissue). Alternatively (or in addition), elevated ploidy may enhance desiccation tolerance simply by increasing the abundance and volume of important defensive and protective compounds such as ELIP or LEA proteins. Although simply being polyploid is unlikely to be related to the evolution of desiccation tolerance, our data suggest that polyploidy sets the stage for rapid post-polyploidization evolution and could be a mechanism for enhancing existing tolerance mechanisms and indirectly facilitating local adaptation.

Broadly, we describe two distinct ecotypes of *M. caffra* that exhibit consistent differences in morphological, reproductive, and desiccation tolerance traits in both field and common growth conditions (Fig. 2). Plant phenotypes varied along the environmental gradient of elevation, temperature, and water availability as predicted. We observed improved recovery outcomes in plants from the driest sites compared with plants from more mesic sites (Fig. 3). These data hint at local adaptation and suggest that strong selective pressures in arid environments favor the evolution of enhanced desiccation tolerance, possibly mediated by changes in ploidy. In contrast, plants from mesic sites displayed reduced desiccation tolerance, a possible consequence of relaxed selective pressure on desiccation tolerance in these wetter environments. Because these data were generated from plants cultivated in common conditions, they suggest that genetic, rather than plastic, differences between populations drive variation in recovery from desiccation. Similar patterns have been documented in other plant species (Marks *et al.*, 2016, 2019a; Wang *et al.*, 2018), supporting the notion that adaptation to different moisture regimes can lead to contrasting desiccation tolerance levels (Gutschick and BassiriRad, 2003). However, the ranges of these two ecotypes are not fully quantified, and parallel assessments at sites where both ecotypes occur together could provide additional insight into the complex interplay between environmental differences, ploidy, and desiccation tolerance in *M. caffra.*

We detected complex trade-offs between desiccation tolerance, reproduction, and morphology. Differences in reproductive allocation across the study area trended in the opposite direction to desiccation tolerance traits, with more reproductive plants occurring in the most mesic sites (Fig. 2). Variation in ploidy levels may contribute to the observed reproductive differences, as polyploidy can interfere with meiotic processes that are essential for sexual reproduction and thereby result in reduced seed and panicle number (Comai, 2005; Herben *et al.*, 2017), as observed here. In addition to the effects of polyploidy, reproductive differences across populations could be driven by selection against seed production in xeric sites due to frequent droughts that interrupt floral development, or a trade-off between vegetative and reproductive resource allocation. There may also be a trade-off between desiccation tolerance and reproduction, as has been suggested in the liverwort *Marchantia inflexa* (Marks *et al.*, 2019a, b, 2020). The differences in reproduction observed here parallel findings in the eudicot resurrection plant *Myrothamnus flabellifolia,* which was sampled along the same environmental gradient (Marks *et al.*, 2022). However, in *M. flabellifolia*, desiccation tolerance is maintained at high levels at all sites (Marks *et al.*, 2022), suggesting that trade-offs between desiccation tolerance and reproduction are not universal in resurrection plants.

Our data provide intriguing evidence that desiccation tolerance may be mediated by changes in ploidy and highlight the significance of polyploidy as a potential driver of local adaptation. Taken together, these data support the existence of two diverging ecotypes that occur along an environmental gradient—a small reproductive ecotype from high elevation mesic sites, and a larger but less reproductive ecotype from low elevation xeric sites. These complex interactions illustrate how evolutionary processes associated with genome duplication might shape a plant’s ability to thrive under abiotic stress. Further investigations of specific molecular mechanisms underlying these phenomena will provide insight into the interplay between polyploidy, desiccation tolerance, and local adaptation. We suggest that *M. caffra* is a promising system for exploring the role of polyploidy in facilitating the rapid evolution of desiccation tolerance.

## Supplementary data

The following supplementary data are available at *JXB* online.

Table S1. Geo coordinates, mean annual precipitation and temperature, and elevation of collection sites.

Fig. S1. K-mer analyses of *Microchloa caffra* whole-genome sequencing data.

Fig. S2. Physiological characterization of field drying time course.

Fig. S3. Transmission electron micrographs of mesophyll cells.

Appendix S1. Detailed flow cytometry results.

Dataset S1. Morphological, physiological, and environmental data in excel notebook.

erae126_suppl_Supplementary_Appendix_S1

erae126_suppl_Supplementary_Dataset_S1

erae126_suppl_Supplementary_Figure_S1-S3_Table_S1

## Data Availability

Data associated with this study are provided as supplementary files.
