# Supplementary material for: Higher order polyploids exhibit enhanced desiccation tolerance in the grass Microchloa caffra
Source: J Exp Bot. 2024 Mar 21;75(11):3612–23. doi: 10.1093/jxb/erae126 (PMC11156804; doi:10.1093/jxb/erae126)
Supplement: erae126_suppl_Supplementary_Appendix_S1 [file erae126_suppl_supplementary_appendix_s1.pdf]

APPENDIX S1

Higher order polyploids exhibit enhanced desiccation tolerance in the grass *Microchloa caffra*

Rose A. Marks[1,2,3], Paula Delgado[3], Givemore Munashe Makonya[3,4], Keren Cooper[3], Robert VanBuren[1,2], and Jill M. Farrant[3]

Controls- 2x diploid control is red, 4x tetraploid is blue, 3x is triploid pink. Hosta is Green. Sample ID is above peak profile.

A1 - BK-BR-B ☑ Show Statistics

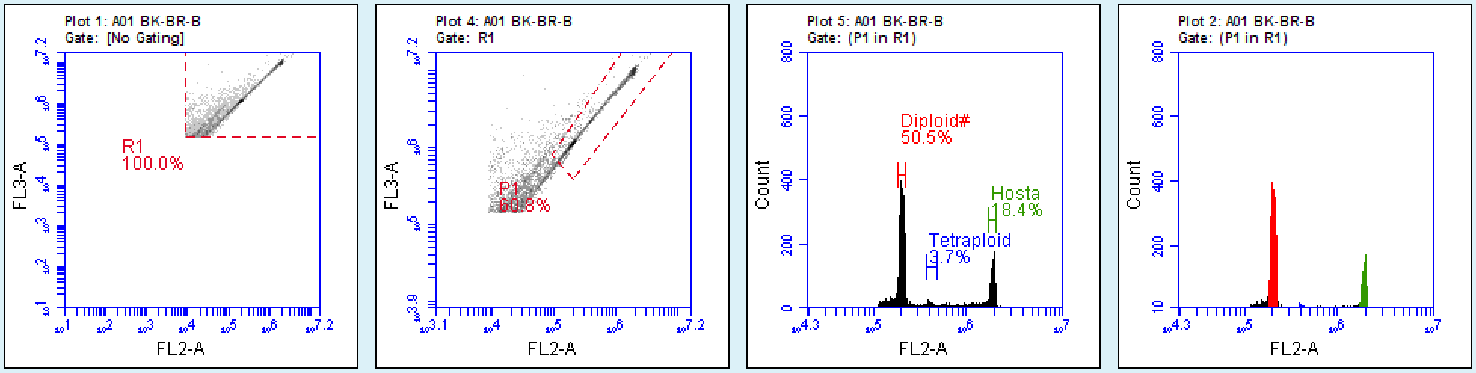

Plot 1: A01 BK-BR-B	Count	Events / µL	% of This Plot	% of All	Mean FL2-A	Mean FL3-A	CV FL2-A	CV FL3-A	Median FL2-A	Median FL3-A
All	5,759	83	100.00%	100.00%	417,696.81	2,266,636.92	157.47%	141.60%		
R1	5,759	83	100.00%	100.00%	417,696.81	2,266,636.92	157.47%	141.60%		

Plot 4: A01 BK-BR-B Gated on R1	Count	Events / µL	% of This Plot	% of All	Mean FL2-A	Mean FL3-A	CV FL2-A	CV FL3-A	Median FL2-A	Median FL3-A
This Plot	5,759	83	100.00%	100.00%	417,696.81	2,266,636.92	157.47%	141.60%		
P1	3,500	51	60.77%	60.77%	658,535.61	3,413,132.03	113.90%	106.85%		

Plot 5: A01 BK-BR-B Gated on (P1 in R1)	Count	Events / µL	% of This Plot	% of All	Mean FL2-A	CV FL2-A	Median FL2-A
This Plot	3,500	51	100.00%	60.77%	658,535.61	113.90%	
Diploid# (177,298.0 /...	1,769	26	50.54%	30.72%	200,735.73	5.35%	
Tetraploid (363,792....	129	2	3.69%	2.24%	415,294.91	8.00%	
Hosta (1,715,701.0 /...	644	9	18.40%	11.18%	1,959,006.03	4.94%	

Plot 2: A01 BK-BR-B Gated on (P1 in R1)	Count	Events / µL	% of This Plot	% of All	Mean FL2-A	CV FL2-A	Median FL2-A
This Plot	3,500	51	100.00%	60.77%	658,535.61	113.90%	

Hosta 'Praying Hands' genome size of 23.34 Gb (2n DNA content).

Sample calculation = 23.34 Gb Hosta 'Praying Hands' genome x (200,735.73/ 1,959,006.03) = 2.39 Gb (2n DNA content)

Microchloa caffra **BK-BR-B genome size of 2.39 Gb (2C DNA content).**

A2 - BK-BR-C ☑ Show Statistics

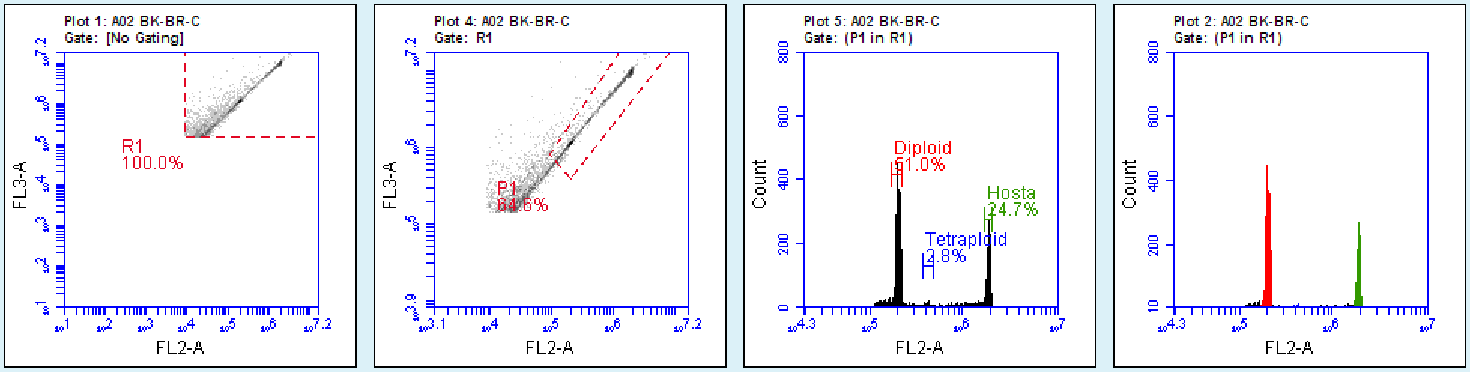

Plot 1: A02 BK-BR-C	Count	Events / µL	% of This Plot	% of All	Mean FL2-A	Mean FL3-A	CV FL2-A	CV FL3-A	Median FL2-A	Median FL3-A
All	5,416	64	100.00%	100.00%	496,967.50	2,613,490.47	142.77%	132.53%		
R1	5,416	64	100.00%	100.00%	496,967.50	2,613,490.47	142.77%	132.53%		

Plot 4: A02 BK-BR-C Gated on R1	Count	Events / µL	% of This Plot	% of All	Mean FL2-A	Mean FL3-A	CV FL2-A	CV FL3-A	Median FL2-A	Median FL3-A
This Plot	5,416	64	100.00%	100.00%	496,967.50	2,613,490.47	142.77%	132.53%		
P1	3,500	42	64.62%	64.62%	744,625.90	3,809,022.02	104.43%	99.15%		

Plot 5: A02 BK-BR-C Gated on (P1 in R1)	Count	Events / µL	% of This Plot	% of All	Mean FL2-A	CV FL2-A	Median FL2-A
This Plot	3,500	42	100.00%	64.62%	744,625.90	104.43%	
Diploid (164,378.0 / 2…	1,786	21	51.03%	32.98%	199,677.04	5.54%	
Tetraploid (363,792.0…	97	1	2.77%	1.79%	420,042.21	7.57%	
Hosta (1,715,701.0 / …	864	10	24.69%	15.95%	1,948,862.87	4.32%	

Plot 2: A02 BK-BR-C Gated on (P1 in R1)	Count	Events / µL	% of This Plot	% of All	Mean FL2-A	CV FL2-A	Median FL2-A
This Plot	3,500	42	100.00%	64.62%	744,625.90	104.43%	

Hosta ‘Praying Hands’ genome size of 23.34 Gb (2n DNA content).

Sample calculation = 23.34 Gb Hosta ‘Praying Hands’ genome x (199,677.04/ 1,948,862.87) = 2.39 Gb (2n DNA content)

***Microchloa caffra* BK-BR-C genome size of 2.39 Gb (2C DNA content).**

A3 - BK-DI-A ☑ Show Statistics

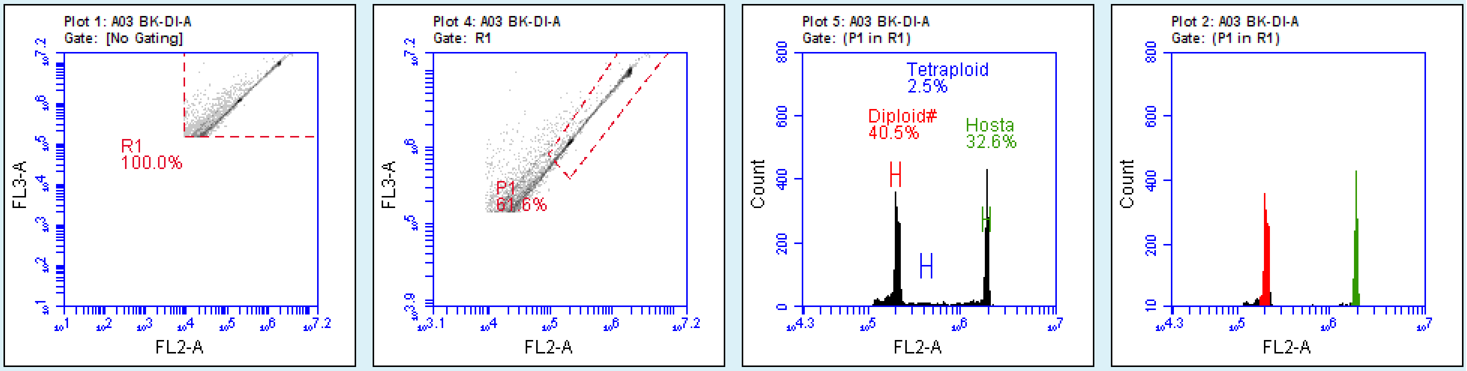

Plot 1: A03 BK-DI-A	Count	Events / µL	% of This Plot	% of All	Mean FL2-A	Mean FL3-A	CV FL2-A	CV FL3-A	Median FL2-A	Median FL3-A
All	5,678	68	100.00%	100.00%	568,621.96	2,950,250.19	138.25%	127.55%		
R1	5,678	68	100.00%	100.00%	568,621.96	2,950,250.19	138.25%	127.55%		

Plot 4: A03 BK-DI-A Gated on R1	Count	Events / µL	% of This Plot	% of All	Mean FL2-A	Mean FL3-A	CV FL2-A	CV FL3-A	Median FL2-A	Median FL3-A
This Plot	5,678	68	100.00%	100.00%	568,621.96	2,950,250.19	138.25%	127.55%		
P1	3,500	42	61.64%	61.64%	895,419.76	4,524,456.92	94.98%	89.29%		

Plot 5: A03 BK-DI-A Gated on (P1 in R1)	Count	Events / µL	% of This Plot	% of All	Mean FL2-A	CV FL2-A	Median FL2-A
This Plot	3,500	42	100.00%	61.64%	895,419.76	94.98%	
Diploid# (170,716.0 ...	1,418	17	40.51%	24.97%	202,189.99	5.59%	
Tetraploid (363,792....	87	1	2.49%	1.53%	420,461.26	8.26%	
Hosta (1,715,701.0 ...	1,142	14	32.63%	20.11%	1,952,148.25	3.94%	

Plot 2: A03 BK-DI-A Gated on (P1 in R1)	Count	Events / µL	% of This Plot	% of All	Mean FL2-A	CV FL2-A	Median FL2-A
This Plot	3,500	42	100.00%	61.64%	895,419.76	94.98%	

Hosta ‘Praying Hands’ genome size of 23.34 Gb (2n DNA content).

Sample calculation = 23.34 Gb Hosta ‘Praying Hands’ genome x (202,189.99/ 1,952,148.25) = 2.42 Gb (2n DNA content)

Microchloa caffra **BK-DI-A genome size of 2.42 Gb (2C DNA content).**

A4 - BK-DI-B ☑ Show Statistics

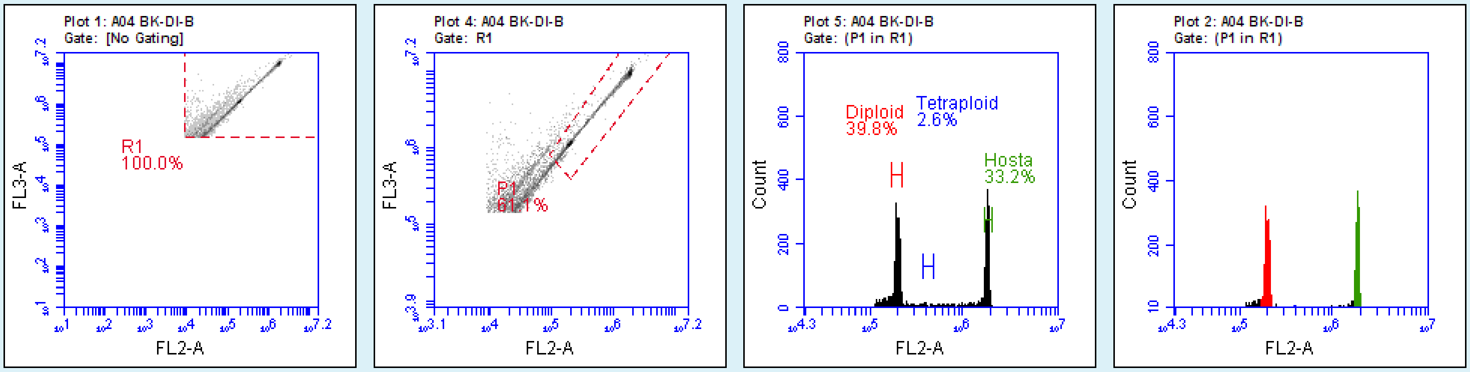

Plot 1: A04 BK-DI-B	Count	Events / µL	% of This Plot	% of All	Mean FL2-A	Mean FL3-A	CV FL2-A	CV FL3-A	Median FL2-A	Median FL3-A
All	5,731	73	100.00%	100.00%	556,347.87	2,952,879.69	134.76%	125.68%		
R1	5,731	73	100.00%	100.00%	556,347.87	2,952,879.69	134.76%	125.68%		

Plot 4: A04 BK-DI-B Gated on R1	Count	Events / µL	% of This Plot	% of All	Mean FL2-A	Mean FL3-A	CV FL2-A	CV FL3-A	Median FL2-A	Median FL3-A
This Plot	5,731	73	100.00%	100.00%	556,347.87	2,952,879.69	134.76%	125.68%		
P1	3,500	44	61.07%	61.07%	882,983.89	4,563,455.69	91.00%	87.00%		

Plot 5: A04 BK-DI-B Gated on (P1 in R1)	Count	Events / µL	% of This Plot	% of All	Mean FL2-A	CV FL2-A	Median FL2-A
This Plot	3,500	44	100.00%	61.07%	882,983.89	91.00%	
Diploid (164,378.0 / ...	1,392	18	39.77%	24.29%	195,053.42	5.89%	
Tetraploid (363,792....	92	1	2.63%	1.61%	416,921.28	9.10%	
Hosta (1,715,701.0 ...	1,162	15	33.20%	20.28%	1,883,039.80	3.97%	

Plot 2: A04 BK-DI-B Gated on (P1 in R1)	Count	Events / µL	% of This Plot	% of All	Mean FL2-A	CV FL2-A	Median FL2-A
This Plot	3,499	44	100.00%	61.05%	881,583.49	90.67%	

Hosta ‘Praying Hands’ genome size of 23.34 Gb (2n DNA content).

Sample calculation = 23.34 Gb Hosta ‘Praying Hands’ genome x (195,053.42/ 1,883,039.80) = 2.42 Gb (2n DNA content)

***Microchloa caffra* BK-DI-B genome size of 2.42 Gb (2C DNA content).**

A5 - BK-LK-C ☑ Show Statistics

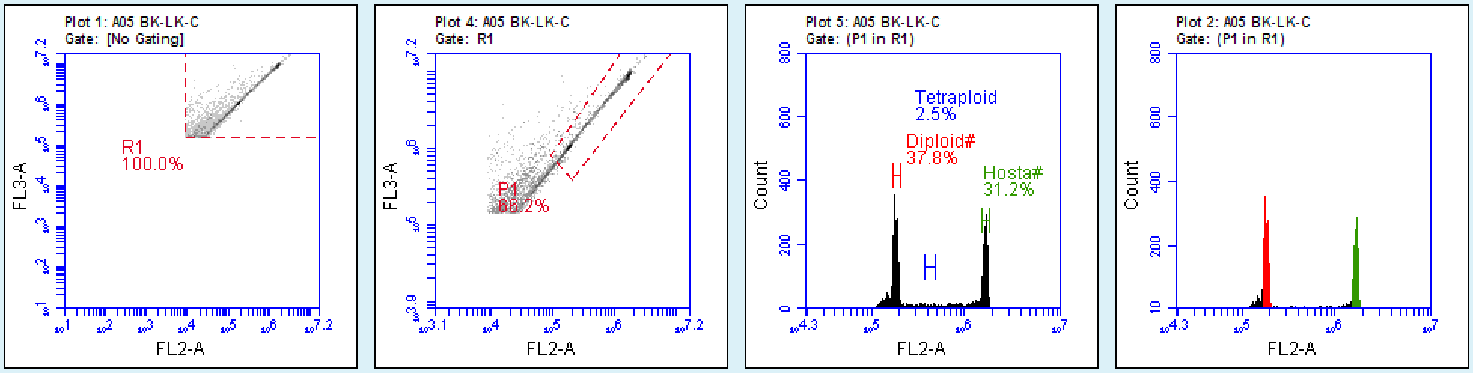

Plot 1: A05 BK-LK-C	Count	Events / µL	% of This Plot	% of All	Mean FL2-A	Mean FL3-A	CV FL2-A	CV FL3-A	Median FL2-A	Median FL3-A
All	5,286	102	100.00%	100.00%	540,154.62	2,923,226.83	127.83%	119.49%		
R1	5,286	102	100.00%	100.00%	540,154.62	2,923,226.83	127.83%	119.49%		

Plot 4: A05 BK-LK-C Gated on R1	Count	Events / µL	% of This Plot	% of All	Mean FL2-A	Mean FL3-A	CV FL2-A	CV FL3-A	Median FL2-A	Median FL3-A
This Plot	5,286	102	100.00%	100.00%	540,154.62	2,923,226.83	127.83%	119.49%		
P1	3,500	67	66.21%	66.21%	792,066.24	4,173,930.02	92.02%	88.11%		

Plot 5: A05 BK-LK-C Gated on (P1 in R1)	Count	Events / µL	% of This Plot	% of All	Mean FL2-A	CV FL2-A	Median FL2-A
This Plot	3,500	67	100.00%	66.21%	792,066.24	92.02%	
Diploid# (164,378.0 /…	1,324	25	37.83%	25.05%	180,046.21	5.02%	
Tetraploid (363,792.0…	87	2	2.49%	1.65%	423,783.67	9.13%	
Hosta# (1,531,633.0…	1,093	21	31.23%	20.68%	1,708,888.97	4.83%	

Plot 2: A05 BK-LK-C Gated on (P1 in R1)	Count	Events / µL	% of This Plot	% of All	Mean FL2-A	CV FL2-A	Median FL2-A
This Plot	3,500	67	100.00%	66.21%	792,066.24	92.02%	

Hosta ‘Praying Hands’ genome size of 23.34 Gb (2n DNA content).

Sample calculation = 23.34 Gb Hosta ‘Praying Hands’ genome x (180,046.21/ 1,708,888.97) = 2.46 Gb (2n DNA content)

***Microchloa caffra* BK-LK-C genome size of 2.46 Gb (2C DNA content).**

A6 - BK-UK-A ☑ Show Statistics

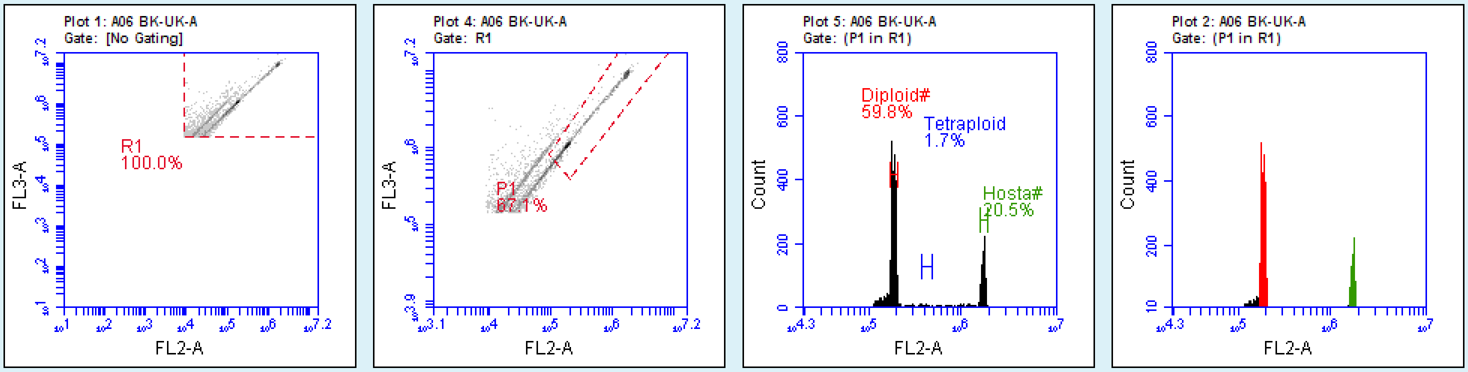

Plot 1: A06 BK-UK-A	Count	Events / µL	% of This Plot	% of All	Mean FL2-A	Mean FL3-A	CV FL2-A	CV FL3-A	Median FL2-A	Median FL3-A
All	5,217	56	100.00%	100.00%	392,322.53	2,149,675.77	150.07%	138.95%		
R1	5,217	56	100.00%	100.00%	392,322.53	2,149,675.77	150.07%	138.95%		

Plot 4: A06 BK-UK-A Gated on R1	Count	Events / µL	% of This Plot	% of All	Mean FL2-A	Mean FL3-A	CV FL2-A	CV FL3-A	Median FL2-A	Median FL3-A
This Plot	5,217	56	100.00%	100.00%	392,322.53	2,149,675.77	150.07%	138.95%		
P1	3,500	38	67.09%	67.09%	561,317.52	2,985,475.48	116.73%	111.43%		

Plot 5: A06 BK-UK-A Gated on (P1 in R1)	Count	Events / µL	% of This Plot	% of All	Mean FL2-A	CV FL2-A	Median FL2-A
This Plot	3,500	38	100.00%	67.09%	561,317.52	116.73%	
Diploid# (164,378.0 /…	2,094	23	59.83%	40.14%	183,400.05	4.99%	
Tetraploid (363,792.0…	58	1	1.66%	1.11%	417,204.78	8.88%	
Hosta# (1,590,683.0…	717	8	20.49%	13.74%	1,764,506.90	4.44%	

Plot 2: A06 BK-UK-A Gated on (P1 in R1)	Count	Events / µL	% of This Plot	% of All	Mean FL2-A	CV FL2-A	Median FL2-A
This Plot	3,500	38	100.00%	67.09%	561,317.52	116.73%	

Hosta 'Praying Hands' genome size of 23.34 Gb (2n DNA content).

Sample calculation = 23.34 Gb Hosta 'Praying Hands' genome x (183,400.05/ 1,764,506.90) = 2.43 Gb (2n DNA content)

Microchloa caffra **BK-UK-A genome size of 2.43 Gb (2C DNA content).**

B1 - BK-UK-B ☑ Show Statistics

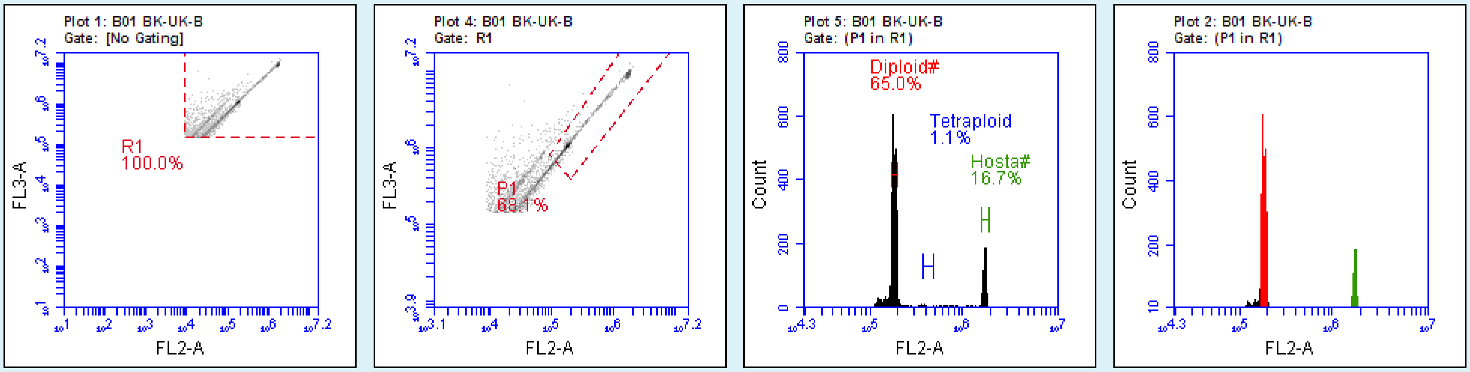

Plot 1: B01 BK-UK-B	Count	Events / µL	% of This Plot	% of All	Mean FL2-A	Mean FL3-A	CV FL2-A	CV FL3-A	Median FL2-A	Median FL3-A
All	5,143	35	100.00%	100.00%	341,769.88	1,910,960.96	157.26%	145.03%		
R1	5,143	35	100.00%	100.00%	341,769.88	1,910,960.96	157.26%	145.03%		

Plot 4: B01 BK-UK-B Gated on R1	Count	Events / µL	% of This Plot	% of All	Mean FL2-A	Mean FL3-A	CV FL2-A	CV FL3-A	Median FL2-A	Median FL3-A
This Plot	5,143	35	100.00%	100.00%	341,769.88	1,910,960.96	157.26%	145.03%		
P1	3,500	24	68.05%	68.05%	481,439.76	2,614,126.37	125.14%	118.92%		

Plot 5: B01 BK-UK-B Gated on (P1 in R1)	Count	Events / µL	% of This Plot	% of All	Mean FL2-A	CV FL2-A	Median FL2-A
This Plot	3,500	24	100.00%	68.05%	481,439.76	125.14%	
Diploid# (164,378.0 /…	2,274	15	64.97%	44.22%	180,285.21	4.84%	
Tetraploid (363,792.…	37	0	1.06%	0.72%	416,286.14	10.48%	
Hosta# (1,590,683.0…	586	4	16.74%	11.39%	1,760,196.42	3.88%	

Plot 2: B01 BK-UK-B Gated on (P1 in R1)	Count	Events / µL	% of This Plot	% of All	Mean FL2-A	CV FL2-A	Median FL2-A
This Plot	3,500	24	100.00%	68.05%	481,439.76	125.14%	

Hosta ‘Praying Hands’ genome size of 23.34 Gb (2n DNA content).

Sample calculation = 23.34 Gb Hosta ‘Praying Hands’ genome x (180,285.21/ 1,760,196.42) = 2.39 Gb (2n DNA content)

Microchloa caffra **BK-UK-B genome size of 2.39 Gb (2C DNA content).**

B2 - Mtn-Sis-A ☑ Show Statistics

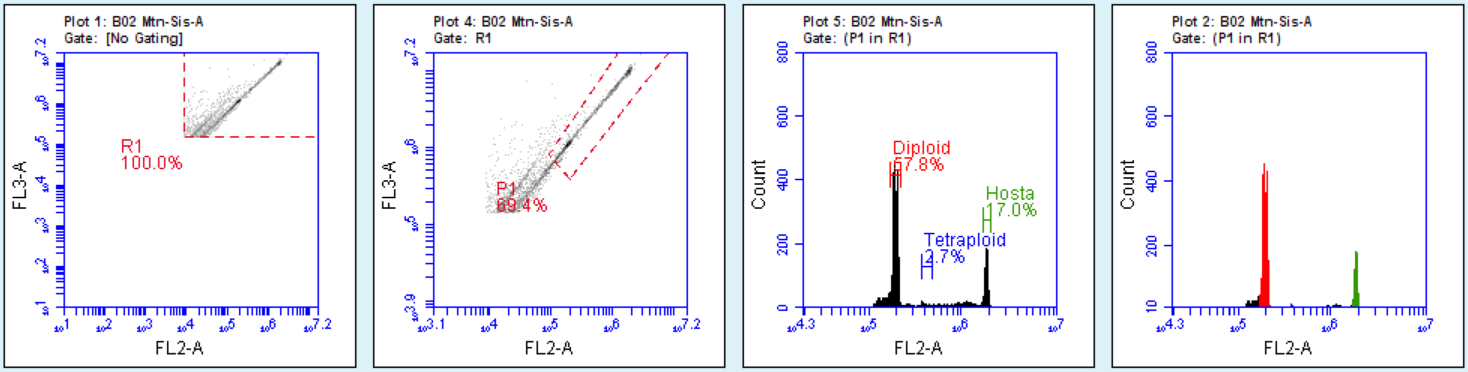

Plot 1: B02 Mtn-Sis-A	Count	Events / µL	% of This Plot	% of All	Mean FL2-A	Mean FL3-A	CV FL2-A	CV FL3-A	Median FL2-A	Median FL3-A
All	5,044	37	100.00%	100.00%	416,994.53	2,283,258.51	146.21%	136.68%		
R1	5,044	37	100.00%	100.00%	416,994.53	2,283,258.51	146.21%	136.68%		

Plot 4: B02 Mtn-Sis-A Gated on R1	Count	Events / µL	% of This Plot	% of All	Mean FL2-A	Mean FL3-A	CV FL2-A	CV FL3-A	Median FL2-A	Median FL3-A
This Plot	5,044	37	100.00%	100.00%	416,994.53	2,283,258.51	146.21%	136.68%		
P1	3,500	26	69.39%	69.39%	581,513.71	3,110,473.73	114.93%	109.82%		

Plot 5: B02 Mtn-Sis-A Gated on (P1 in R1)	Count	Events / µL	% of This Plot	% of All	Mean FL2-A	CV FL2-A	Median FL2-A
This Plot	3,500	26	100.00%	69.39%	581,513.71	114.93%	
Diploid (164,378.0 / 22…	2,023	15	57.80%	40.11%	191,486.72	5.64%	
Tetraploid (363,792.0 /…	94	1	2.69%	1.86%	411,251.63	8.61%	
Hosta (1,715,701.0 / 2…	595	4	17.00%	11.80%	1,900,520.42	4.05%	

Plot 2: B02 Mtn-Sis-A Gated on (P1 in R1)	Count	Events / µL	% of This Plot	% of All	Mean FL2-A	CV FL2-A	Median FL2-A
This Plot	3,500	26	100.00%	69.39%	581,513.71	114.93%	

Hosta 'Praying Hands' genome size of 23.34 Gb (2n DNA content).

Sample calculation = 23.34 Gb Hosta 'Praying Hands' genome x (191,486.72/ 1,900,520.42) = 2.35 Gb (2n DNA content)

***Microchloa caffra* Mtn-Sis-A genome size of 2.35 Gb (2C DNA content).**

B3 - Mtn-Sis-B ☑ Show Statistics

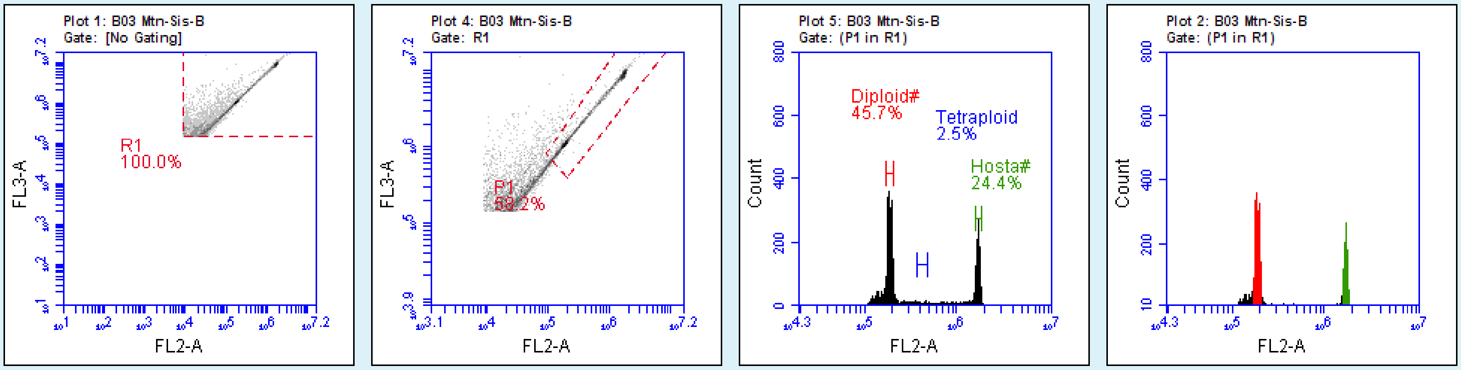

Plot 1: B03 Mtn-Sis-B	Count	Events / µL	% of This Plot	% of All	Mean FL2-A	Mean FL3-A	CV FL2-A	CV FL3-A	Median FL2-A	Median FL3-A
All	6,013	99	100.00%	100.00%	409,427.81	2,235,045.82	154.97%	139.12%		
R1	6,013	99	100.00%	100.00%	409,427.81	2,235,045.82	154.97%	139.12%		

Plot 4: B03 Mtn-Sis-B Gated on R1	Count	Events / µL	% of This Plot	% of All	Mean FL2-A	Mean FL3-A	CV FL2-A	CV FL3-A	Median FL2-A	Median FL3-A
This Plot	6,013	99	100.00%	100.00%	409,427.81	2,235,045.82	154.97%	139.12%		
P1	3,500	57	58.21%	58.21%	670,022.04	3,522,432.77	104.78%	99.70%		

Plot 5: B03 Mtn-Sis-B Gated on (P1 in R1)	Count	Events / µL	% of This Plot	% of All	Mean FL2-A	CV FL2-A	Median FL2-A
This Plot	3,500	57	100.00%	58.21%	670,022.04	104.78%	
Diploid# (164,378.0 / 2...	1,599	26	45.69%	26.59%	184,828.14	5.34%	
Tetraploid (363,792.0 /...	89	1	2.54%	1.48%	424,835.56	8.96%	
Hosta# (1,590,683.0 / ...	855	14	24.43%	14.22%	1,738,448.67	4.24%	

Plot 2: B03 Mtn-Sis-B Gated on (P1 in R1)	Count	Events / µL	% of This Plot	% of All	Mean FL2-A	CV FL2-A	Median FL2-A
This Plot	3,500	57	100.00%	58.21%	670,022.04	104.78%	

Hosta 'Praying Hands' genome size of 23.34 Gb (2n DNA content).

Sample calculation = 23.34 Gb Hosta 'Praying Hands' genome x (184,828.14/ 1,738,448.67) = 2.48 Gb (2n DNA content)

***Microchloa caffra* Mtn-Sis-B genome size of 2.48 Gb (2C DNA content).**

B4 - SS-BR-A ☑ Show Statistics

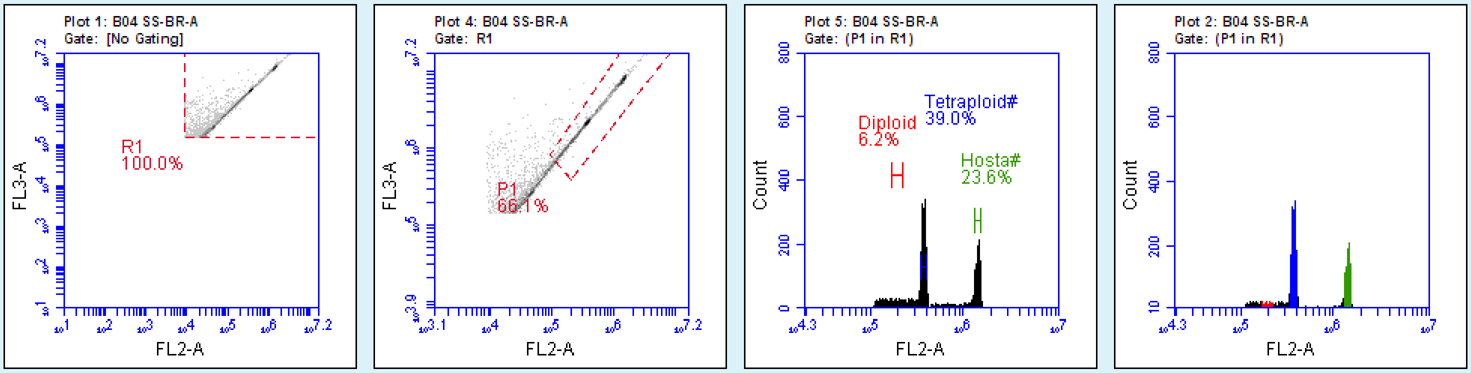

Plot 1: B04 SS-BR-A	Count	Events / µL	% of This Plot	% of All	Mean FL2-A	Mean FL3-A	CV FL2-A	CV FL3-A	Median FL2-A	Median FL3-A
All	5,294	90	100.00%	100.00%	454,761.63	2,567,482.19	113.82%	107.45%		
R1	5,294	90	100.00%	100.00%	454,761.63	2,567,482.19	113.82%	107.45%		

Plot 4: B04 SS-BR-A Gated on R1	Count	Events / µL	% of This Plot	% of All	Mean FL2-A	Mean FL3-A	CV FL2-A	CV FL3-A	Median FL2-A	Median FL3-A
This Plot	5,294	90	100.00%	100.00%	454,761.63	2,567,482.19	113.82%	107.45%		
P1	3,500	59	66.11%	66.11%	665,042.21	3,689,914.29	78.76%	75.28%		

Plot 5: B04 SS-BR-A Gated on (P1 in R1)	Count	Events / µL	% of This Plot	% of All	Mean FL2-A	CV FL2-A	Median FL2-A
This Plot	3,500	59	100.00%	66.11%	665,042.21	78.76%	
Diploid (164,378.0 / 2…	216	4	6.17%	4.08%	191,975.69	8.84%	
Tetraploid# (337,284…	1,364	23	38.97%	25.77%	371,752.75	4.73%	
Hosta# (1,316,555.0…	827	14	23.63%	15.62%	1,457,054.84	4.88%	

Plot 2: B04 SS-BR-A Gated on (P1 in R1)	Count	Events / µL	% of This Plot	% of All	Mean FL2-A	CV FL2-A	Median FL2-A
This Plot	3,500	59	100.00%	66.11%	665,042.21	78.76%	

Hosta ‘Praying Hands’ genome size of 23.34 Gb (2n DNA content).

Sample calculation = 23.34 Gb Hosta ‘Praying Hands’ genome x (371,752.75/ 1,457,054.84) = 5.95 Gb (2n DNA content)

Microchloa caffra **SS-BR-A genome size of 5.95 Gb (2C DNA content).**
Probably 5x or 6x

B5 - SS-BR-C ☑ Show Statistics

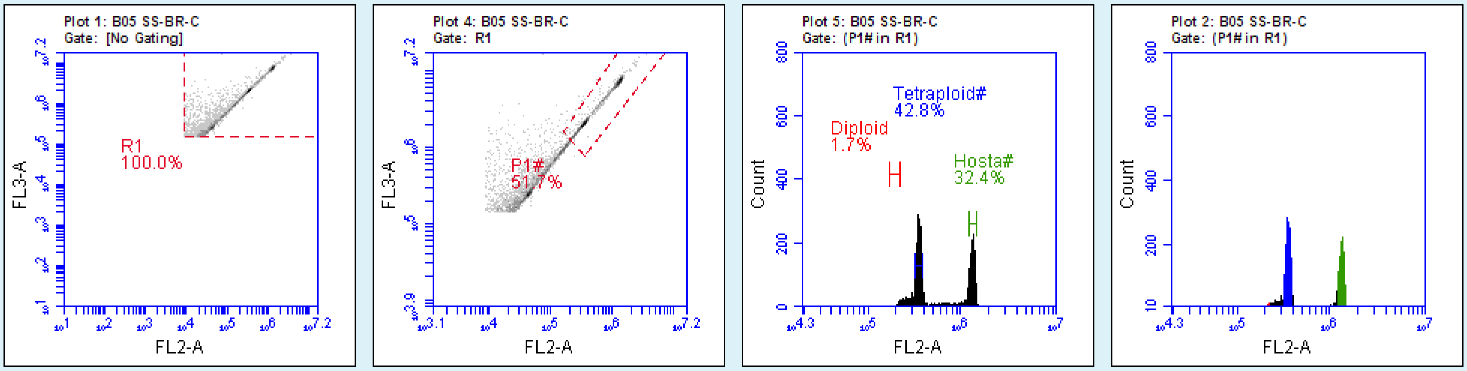

Plot 1: B05 SS-BR-C	Count	Events / µL	% of This Plot	% of All	Mean FL2-A	Mean FL3-A	CV FL2-A	CV FL3-A	Median FL2-A	Median FL3-A
All	5,908	155	100.00%	100.00%	415,772.91	2,351,836.80	119.95%	112.60%		
R1	5,908	155	100.00%	100.00%	415,772.91	2,351,836.80	119.95%	112.60%		

Plot 4: B05 SS-BR-C Gated on R1	Count	Events / µL	% of This Plot	% of All	Mean FL2-A	Mean FL3-A	CV FL2-A	CV FL3-A	Median FL2-A	Median FL3-A
This Plot	5,908	155	100.00%	100.00%	415,772.91	2,351,836.80	119.95%	112.60%		
P1#	3,052	80	51.66%	51.66%	748,106.81	4,121,210.34	66.94%	63.52%		

Plot 5: B05 SS-BR-C Gated on (P1# in R1)	Count	Events / µL	% of This Plot	% of All	Mean FL2-A	CV FL2-A	Median FL2-A
This Plot	3,052	80	100.00%	51.66%	748,106.81	66.94%	
Diploid (164,378.0 / 2…	51	1	1.67%	0.86%	207,391.55	6.27%	
Tetraploid# (312,707.…	1,305	34	42.76%	22.09%	352,965.63	5.52%	
Hosta# (1,220,621.0 …	990	26	32.44%	16.76%	1,364,402.77	5.51%	

Plot 2: B05 SS-BR-C Gated on (P1# in R1)	Count	Events / µL	% of This Plot	% of All	Mean FL2-A	CV FL2-A	Median FL2-A
This Plot	3,052	80	100.00%	51.66%	748,106.81	66.94%	

Hosta ‘Praying Hands’ genome size of 23.34 Gb (2n DNA content).

Sample calculation = 23.34 Gb Hosta ‘Praying Hands’ genome x (352,965.63/ 1,364,402.77) = 6.04 Gb (2n DNA content)

***Microchloa caffra* SS-BR-C genome size of 6.04 Gb (2C DNA content).**

Probably 5x or 6x

B6 - SS-LT-A ☑ Show Statistics

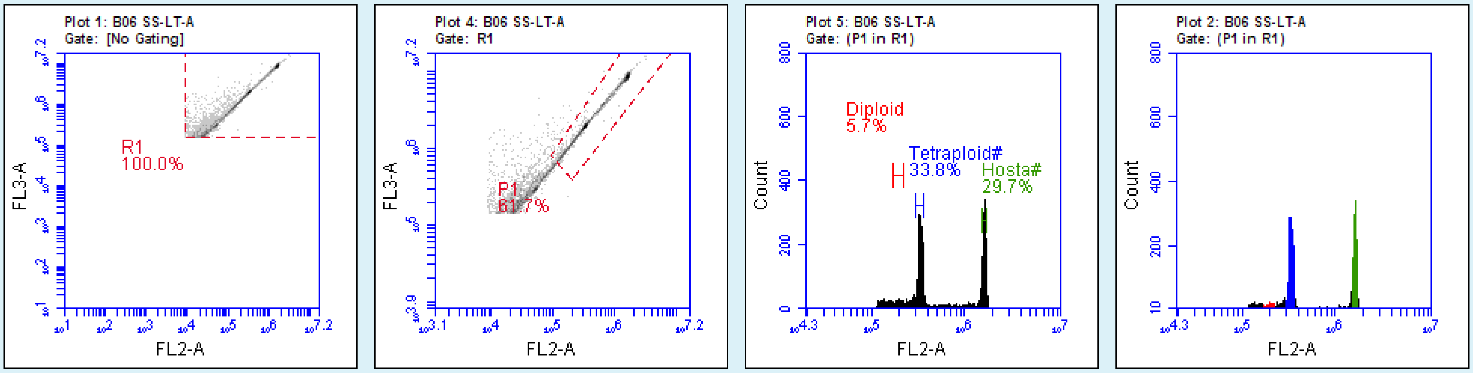

Plot 1: B06 SS-LT-A	Count	Events / µL	% of This Plot	% of All	Mean FL2-A	Mean FL3-A	CV FL2-A	CV FL3-A	Median FL2-A	Median FL3-A
All	5,675	89	100.00%	100.00%	517,871.53	2,790,621.83	121.82%	114.48%		
R1	5,675	89	100.00%	100.00%	517,871.53	2,790,621.83	121.82%	114.48%		

Plot 4: B06 SS-LT-A Gated on R1	Count	Events / µL	% of This Plot	% of All	Mean FL2-A	Mean FL3-A	CV FL2-A	CV FL3-A	Median FL2-A	Median FL3-A
This Plot	5,675	89	100.00%	100.00%	517,871.53	2,790,621.83	121.82%	114.48%		
P1	3,500	55	61.67%	61.67%	814,001.58	4,302,739.78	79.23%	75.28%		

Plot 5: B06 SS-LT-A Gated on (P1 in R1)	Count	Events / µL	% of This Plot	% of All	Mean FL2-A	CV FL2-A	Median FL2-A
This Plot	3,500	55	100.00%	61.67%	814,001.58	79.23%	
Diploid (164,378.0 / 2…	198	3	5.66%	3.49%	193,589.24	8.31%	
Tetraploid# (289,921…	1,183	18	33.80%	20.85%	332,378.33	4.98%	
Hosta# (1,531,633.0…	1,041	16	29.74%	18.34%	1,659,356.50	3.68%	

Plot 2: B06 SS-LT-A Gated on (P1 in R1)	Count	Events / µL	% of This Plot	% of All	Mean FL2-A	CV FL2-A	Median FL2-A
This Plot	3,500	55	100.00%	61.67%	814,001.58	79.23%	

Hosta ‘Praying Hands’ genome size of 23.34 Gb (2n DNA content).

Sample calculation = 23.34 Gb Hosta ‘Praying Hands’ genome x (332,378.33/ 1,659,356.50) = 4.68 Gb (2n DNA content)

***Microchloa caffra* SS-LT-A genome size of 4.68 Gb (2C DNA content).**

C1 - SS-LT-C ☑ Show Statistics

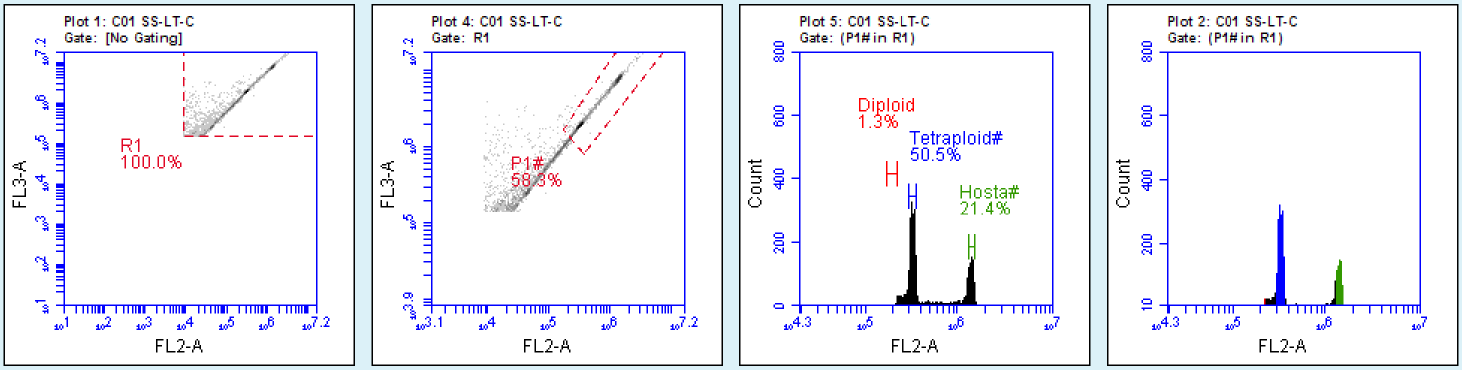

Plot 1: C01 SS-LT-C	Count	Events / µL	% of This Plot	% of All	Mean FL2-A	Mean FL3-A	CV FL2-A	CV FL3-A	Median FL2-A	Median FL3-A
All	5,048	103	100.00%	100.00%	426,997.86	2,421,685.41	116.28%	108.71%		
R1	5,048	103	100.00%	100.00%	426,997.86	2,421,685.41	116.28%	108.71%		

Plot 4: C01 SS-LT-C Gated on R1	Count	Events / µL	% of This Plot	% of All	Mean FL2-A	Mean FL3-A	CV FL2-A	CV FL3-A	Median FL2-A	Median FL3-A
This Plot	5,048	103	100.00%	100.00%	426,997.86	2,421,685.41	116.28%	108.71%		
P1#	2,942	60	58.28%	58.28%	678,362.36	3,762,988.58	76.47%	72.47%		

Plot 5: C01 SS-LT-C Gated on (P1# in R1)	Count	Events / µL	% of This Plot	% of All	Mean FL2-A	CV FL2-A	Median FL2-A
This Plot	2,942	60	100.00%	58.28%	678,362.36	76.47%	
Diploid (164,378.0 / 2...	37	1	1.26%	0.73%	216,635.14	2.87%	
Tetraploid# (289,921....	1,485	30	50.48%	29.42%	326,497.41	5.45%	
Hosta# (1,316,555.0 ...	631	13	21.45%	12.50%	1,441,448.17	4.93%	

Plot 2: C01 SS-LT-C Gated on (P1# in R1)	Count	Events / µL	% of This Plot	% of All	Mean FL2-A	CV FL2-A	Median FL2-A
This Plot	2,942	60	100.00%	58.28%	678,362.36	76.47%	

Hosta ‘Praying Hands’ genome size of 23.34 Gb (2n DNA content).

Sample calculation = 23.34 Gb Hosta ‘Praying Hands’ genome x (326,497.41/ 1,441,448.17) = 5.29 Gb (2n DNA content)

***Microchloa caffra* SS-LT-C genome size of 5.29 Gb (2C DNA content).**

C2 - SS-RV-A ☑ Show Statistics

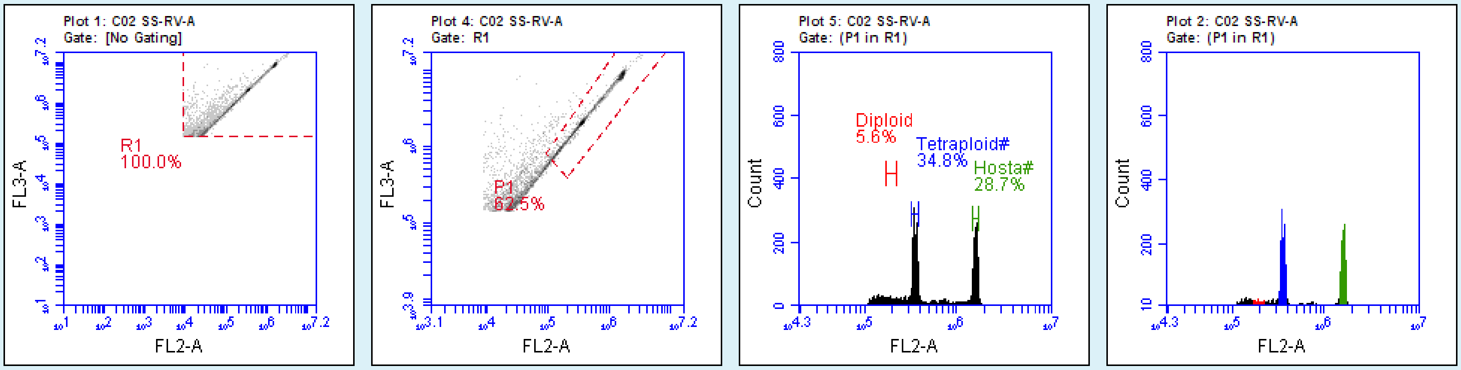

Plot 1: C02 SS-RV-A	Count	Events / µL	% of This Plot	% of All	Mean FL2-A	Mean FL3-A	CV FL2-A	CV FL3-A	Median FL2-A	Median FL3-A
All	5,597	46	100.00%	100.00%	506,309.15	2,832,608.64	122.42%	113.81%		
R1	5,597	46	100.00%	100.00%	506,309.15	2,832,608.64	122.42%	113.81%		

Plot 4: C02 SS-RV-A Gated on R1	Count	Events / µL	% of This Plot	% of All	Mean FL2-A	Mean FL3-A	CV FL2-A	CV FL3-A	Median FL2-A	Median FL3-A
This Plot	5,597	46	100.00%	100.00%	506,309.15	2,832,608.64	122.42%	113.81%		
P1	3,500	28	62.53%	62.53%	784,115.60	4,270,648.90	81.43%	77.14%		

Plot 5: C02 SS-RV-A Gated on (P1 in R1)	Count	Events / µL	% of This Plot	% of All	Mean FL2-A	CV FL2-A	Median FL2-A
This Plot	3,500	28	100.00%	62.53%	784,115.60	81.43%	
Diploid (164,378.0 / 2…	197	2	5.63%	3.52%	191,470.47	8.94%	
Tetraploid# (312,707…	1,219	10	34.83%	21.78%	353,322.09	5.19%	
Hosta# (1,474,775.0 …	1,004	8	28.69%	17.94%	1,630,488.76	4.59%	

Plot 2: C02 SS-RV-A Gated on (P1 in R1)	Count	Events / µL	% of This Plot	% of All	Mean FL2-A	CV FL2-A	Median FL2-A
This Plot	3,500	28	100.00%	62.53%	784,115.60	81.43%	

Hosta ‘Praying Hands’ genome size of 23.34 Gb (2n DNA content).

Sample calculation = 23.34 Gb Hosta ‘Praying Hands’ genome x (353,322.09/ 1.630,488.76) = 5.05 Gb (2n DNA content)

***Microchloa caffra* SS-RV-A genome size of 5.05 Gb (2C DNA content).**

C3 - SS-RV-B ☑ Show Statistics

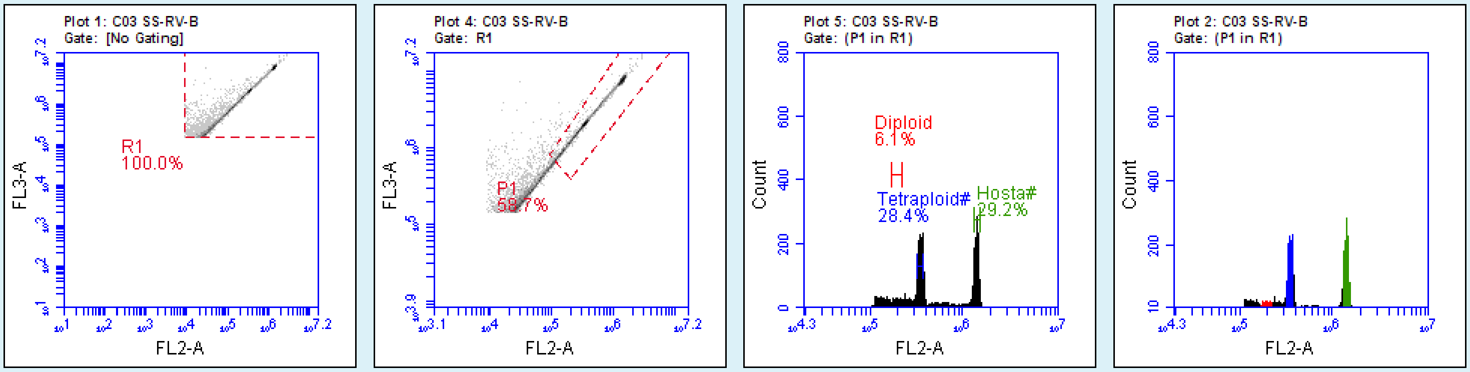

Plot 1: C03 SS-RV-B	Count	Events / µL	% of This Plot	% of All	Mean FL2-A	Mean FL3-A	CV FL2-A	CV FL3-A	Median FL2-A	Median FL3-A
All	5,960	53	100.00%	100.00%	438,651.85	2,479,656.40	122.61%	115.17%		
R1	5,960	53	100.00%	100.00%	438,651.85	2,479,656.40	122.61%	115.17%		

Plot 4: C03 SS-RV-B Gated on R1	Count	Events / µL	% of This Plot	% of All	Mean FL2-A	Mean FL3-A	CV FL2-A	CV FL3-A	Median FL2-A	Median FL3-A
This Plot	5,960	53	100.00%	100.00%	438,651.85	2,479,656.40	122.61%	115.17%		
P1	3,500	31	58.72%	58.72%	715,601.52	3,962,840.14	77.31%	73.36%		

Plot 5: C03 SS-RV-B Gated on (P1 in R1)	Count	Events / µL	% of This Plot	% of All	Mean FL2-A	CV FL2-A	Median FL2-A
This Plot	3,500	31	100.00%	58.72%	715,601.52	77.31%	
Diploid (164,378.0 / 2…	214	2	6.11%	3.59%	193,289.07	8.45%	
Tetraploid# (312,707…	994	9	28.40%	16.68%	348,114.69	4.91%	
Hosta# (1,316,555.0…	1,021	9	29.17%	17.13%	1,438,776.26	4.63%	

Plot 2: C03 SS-RV-B Gated on (P1 in R1)	Count	Events / µL	% of This Plot	% of All	Mean FL2-A	CV FL2-A	Median FL2-A
This Plot	3,500	31	100.00%	58.72%	715,601.52	77.31%	

Hosta ‘Praying Hands’ genome size of 23.34 Gb (2n DNA content).

Sample calculation = 23.34 Gb Hosta ‘Praying Hands’ genome x (348,114.69/ 1,438,776.26) = 5.65 Gb (2n DNA content)

***Microchloa caffra* SS-RV-B genome size of 5.65 Gb (2C DNA content).**

C4 - SS-WC-A ☑ Show Statistics

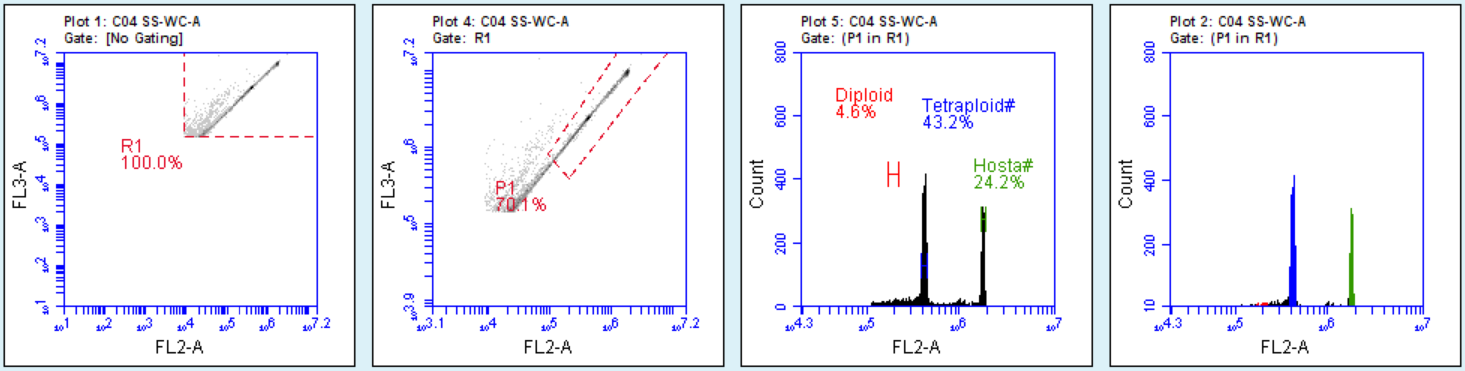

Plot 1: C04 SS-WC-A	Count	Events / µL	% of This Plot	% of All	Mean FL2-A	Mean FL3-A	CV FL2-A	CV FL3-A	Median FL2-A	Median FL3-A
All	4,962	28	100.00%	100.00%	574,881.74	3,120,263.41	111.61%	104.62%		
R1	4,962	28	100.00%	100.00%	574,881.74	3,120,263.41	111.61%	104.62%		

Plot 4: C04 SS-WC-A Gated on R1	Count	Events / µL	% of This Plot	% of All	Mean FL2-A	Mean FL3-A	CV FL2-A	CV FL3-A	Median FL2-A	Median FL3-A
This Plot	4,962	28	100.00%	100.00%	574,881.74	3,120,263.41	111.61%	104.62%		
P1	3,476	20	70.05%	70.05%	803,192.12	4,297,412.01	80.04%	75.30%		

Plot 5: C04 SS-WC-A Gated on (P1 in R1)	Count	Events / µL	% of This Plot	% of All	Mean FL2-A	CV FL2-A	Median FL2-A
This Plot	3,476	20	100.00%	70.05%	803,192.12	80.04%	
Diploid (164,378.0 / 2…	159	1	4.57%	3.20%	193,926.10	8.70%	
Tetraploid# (377,818.…	1,503	8	43.24%	30.29%	417,832.44	4.38%	
Hosta# (1,715,701.0 …	841	5	24.19%	16.95%	1,834,003.78	3.11%	

Plot 2: C04 SS-WC-A Gated on (P1 in R1)	Count	Events / µL	% of This Plot	% of All	Mean FL2-A	CV FL2-A	Median FL2-A
This Plot	3,476	20	100.00%	70.05%	803,192.12	80.04%	

Hosta ‘Praying Hands’ genome size of 23.34 Gb (2n DNA content).

Sample calculation = 23.34 Gb Hosta ‘Praying Hands’ genome x (417,832.44/ 1,834,003.78) = 5.32 Gb (2n DNA content)

***Microchloa caffra* SS-WC-A genome size of 5.32 Gb (2C DNA content).**

C5 - SS-WC-B ☑ Show Statistics

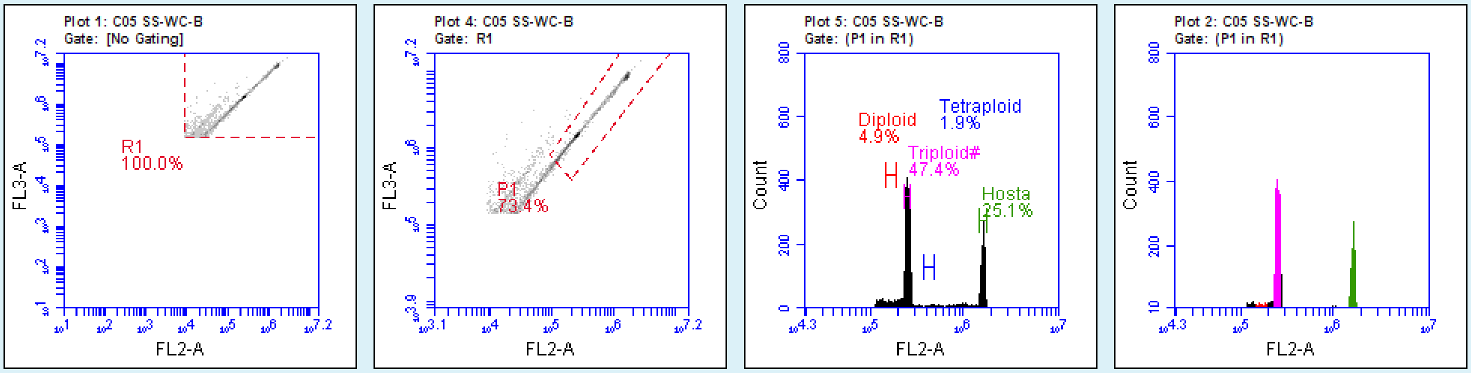

Plot 1: C05 SS-WC-B	Count	Events / µL	% of This Plot	% of All	Mean FL2-A	Mean FL3-A	CV FL2-A	CV FL3-A	Median FL2-A	Median FL3-A
All	4,771	40	100.00%	100.00%	489,088.89	2,645,230.67	121.81%	114.68%		
R1	4,771	40	100.00%	100.00%	489,088.89	2,645,230.67	121.81%	114.68%		

Plot 4: C05 SS-WC-B Gated on R1	Count	Events / µL	% of This Plot	% of All	Mean FL2-A	Mean FL3-A	CV FL2-A	CV FL3-A	Median FL2-A	Median FL3-A
This Plot	4,771	40	100.00%	100.00%	489,088.89	2,645,230.67	121.81%	114.68%		
P1	3,500	29	73.36%	73.36%	650,614.51	3,475,383.41	95.44%	90.65%		

Plot 5: C05 SS-WC-B Gated on (P1 in R1)	Count	Events / µL	% of This Plot	% of All	Mean FL2-A	CV FL2-A	Median FL2-A
This Plot	3,500	29	100.00%	73.36%	650,614.51	95.44%	
Diploid (141,296.0 / 1…	171	1	4.89%	3.58%	165,690.29	8.53%	
Tetraploid (363,792.0 …	68	1	1.94%	1.43%	439,363.50	7.95%	
Hosta (1,474,775.0 / …	877	7	25.06%	18.38%	1,658,950.51	4.39%	
Triploid# (222,473.0 /…	1,659	14	47.40%	34.77%	247,767.07	4.65%	

Plot 2: C05 SS-WC-B Gated on (P1 in R1)	Count	Events / µL	% of This Plot	% of All	Mean FL2-A	CV FL2-A	Median FL2-A
This Plot	3,500	29	100.00%	73.36%	650,614.51	95.44%	

Hosta ‘Praying Hands’ genome size of 23.34 Gb (2n DNA content).

Sample calculation = 23.34 Gb Hosta ‘Praying Hands’ genome x (247,767.07/ 1,658,950.51) = 3.49 Gb (2n DNA content)

***Microchloa caffra* SS-WC-B genome size of 3.49 Gb (2C DNA content).**

Sample is triploid

C6 - VL-FALLS-A ☑ Show Statistics

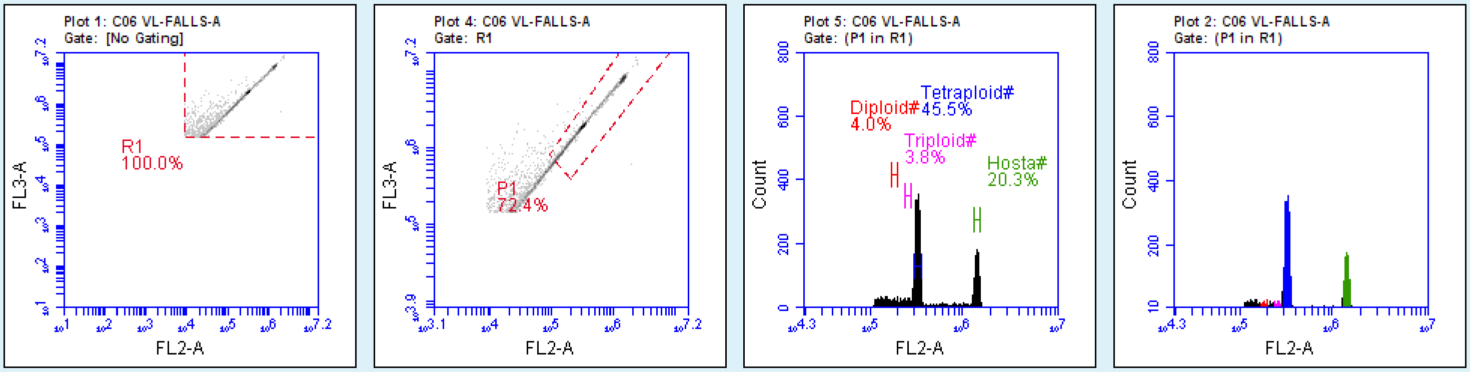

Plot 1: C06 VL-FALLS-A	Count	Events / µL	% of This Plot	% of All	Mean FL2-A	Mean FL3-A	CV FL2-A	CV FL3-A	Median FL2-A	Median FL3-A
All	4,836	35	100.00%	100.00%	440,548.08	2,500,972.50	111.40%	105.34%		
R1	4,836	35	100.00%	100.00%	440,548.08	2,500,972.50	111.40%	105.34%		

Plot 4: C06 VL-FALLS-A Gated on R1	Count	Events / µL	% of This Plot	% of All	Mean FL2-A	Mean FL3-A	CV FL2-A	CV FL3-A	Median FL2-A	Median FL3-A
This Plot	4,836	35	100.00%	100.00%	440,548.08	2,500,972.50	111.40%	105.34%		
P1	3,500	26	72.37%	72.37%	590,094.79	3,314,420.24	84.81%	80.73%		

Plot 5: C06 VL-FALLS-A Gated on (P1 in R1)	Count	Events / µL	% of This Plot	% of All	Mean FL2-A	CV FL2-A	Median FL2-A
This Plot	3,500	26	100.00%	72.37%	590,094.79	84.81%	
Diploid# (164,378.0 / 198…	139	1	3.97%	2.87%	182,064.50	5.29%	
Tetraploid# (289,921.0 / …	1,593	12	45.51%	32.94%	324,377.65	5.25%	
Hosta# (1,316,555.0 / 1,…	710	5	20.29%	14.68%	1,450,200.41	4.64%	
Triploid# (231,050.0 / 279…	133	1	3.80%	2.75%	253,578.45	5.48%	

Plot 2: C06 VL-FALLS-A Gated on (P1 in R1)	Count	Events / µL	% of This Plot	% of All	Mean FL2-A	CV FL2-A	Median FL2-A
This Plot	3,500	26	100.00%	72.37%	590,094.79	84.81%	

Hosta ‘Praying Hands’ genome size of 23.34 Gb (2n DNA content).

Sample calculation = 23.34 Gb Hosta ‘Praying Hands’ genome x (324,377.65/ 1,450,200.41) = 5.22 Gb (2n DNA content)

***Microchloa caffra* VL-FALLS-A genome size of 5.22 Gb (2C DNA content).**

D1 - VL-FALLS-C ☑ Show Statistics

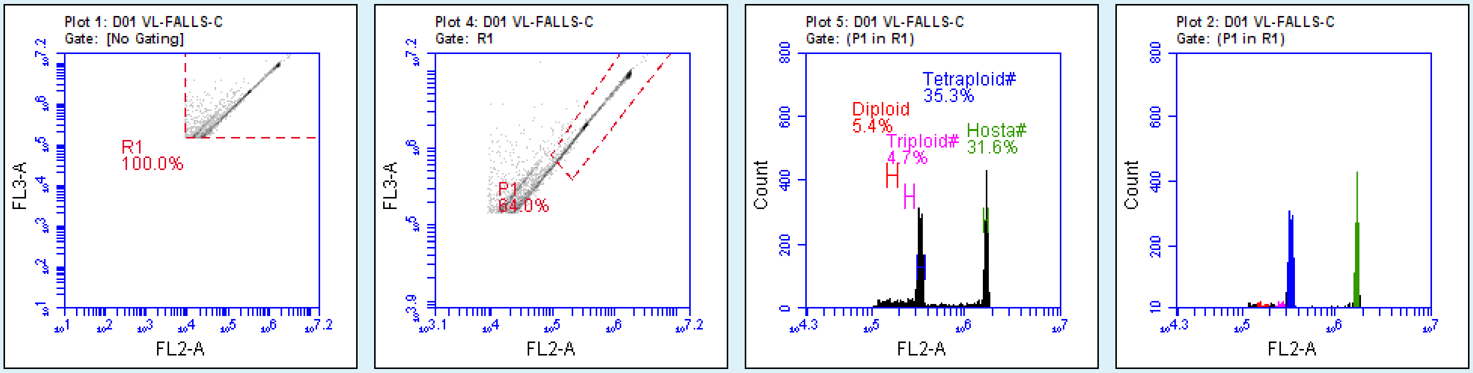

Plot 1: D01 VL-FALLS-C	Count	Events / µL	% of This Plot	% of All	Mean FL2-A	Mean FL3-A	CV FL2-A	CV FL3-A	Median FL2-A	Median FL3-A
All	5,469	51	100.00%	100.00%	562,645.29	3,024,512.54	120.39%	113.05%		
R1	5,469	51	100.00%	100.00%	562,645.29	3,024,512.54	120.39%	113.05%		

Plot 4: D01 VL-FALLS-C Gated on R1	Count	Events / µL	% of This Plot	% of All	Mean FL2-A	Mean FL3-A	CV FL2-A	CV FL3-A	Median FL2-A	Median FL3-A
This Plot	5,469	51	100.00%	100.00%	562,645.29	3,024,512.54	120.39%	113.05%		
P1	3,500	33	64.00%	64.00%	857,027.38	4,522,791.79	80.47%	76.25%		

Plot 5: D01 VL-FALLS-C Gated on (P1 in R1)	Count	Events / µL	% of This Plot	% of All	Mean FL2-A	CV FL2-A	Median FL2-A
This Plot	3,500	33	100.00%	64.00%	857,027.38	80.47%	
Diploid (141,296.0 / 191,2…	190	2	5.43%	3.47%	163,606.08	9.05%	
Tetraploid# (301,099.0 / …	1,236	12	35.31%	22.60%	331,927.11	4.79%	
Hosta# (1,590,683.0 / 1,…	1,106	10	31.60%	20.22%	1,735,091.23	3.45%	
Triploid# (222,473.0 / 28…	166	2	4.74%	3.04%	257,046.30	7.24%	

Plot 2: D01 VL-FALLS-C Gated on (P1 in R1)	Count	Events / µL	% of This Plot	% of All	Mean FL2-A	CV FL2-A	Median FL2-A
This Plot	3,500	33	100.00%	64.00%	857,027.38	80.47%	

Hosta 'Praying Hands' genome size of 23.34 Gb (2n DNA content).

Sample calculation = 23.34 Gb Hosta 'Praying Hands' genome x (331,927.11/ 1,735,091.23) = 4.46 Gb (2n DNA content)

Microchloa caffra **VL-FALLS-C genome size of 4.46 Gb (2C DNA content).**

D2 - VL-MG-A ☑ Show Statistics

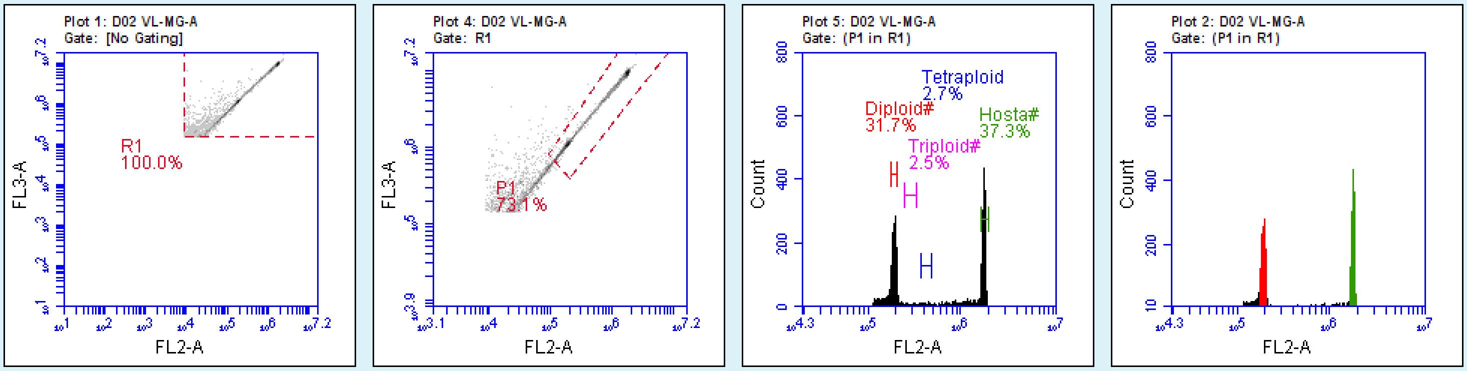

Plot 1: D02 VL-MG-A	Count	Events / µL	% of This Plot	% of All	Mean FL2-A	Mean FL3-A	CV FL2-A	CV FL3-A	Median FL2-A	Median FL3-A
All	4,785	36	100.00%	100.00%	687,143.54	3,649,916.97	111.08%	105.89%		
R1	4,785	36	100.00%	100.00%	687,143.54	3,649,916.97	111.08%	105.89%		

Plot 4: D02 VL-MG-A Gated on R1	Count	Events / µL	% of This Plot	% of All	Mean FL2-A	Mean FL3-A	CV FL2-A	CV FL3-A	Median FL2-A	Median FL3-A
This Plot	4,785	36	100.00%	100.00%	687,143.54	3,649,916.97	111.08%	105.89%		
P1	3,500	26	73.15%	73.15%	922,724.52	4,839,393.87	83.20%	80.05%		

Plot 5: D02 VL-MG-A Gated on (P1 in R1)	Count	Events / µL	% of This Plot	% of All	Mean FL2-A	CV FL2-A	Median FL2-A
This Plot	3,500	26	100.00%	73.15%	922,724.52	83.20%	
Diploid# (170,716.0 / …	1,108	8	31.66%	23.16%	188,893.79	4.74%	
Tetraploid (363,792.0 …	95	1	2.71%	1.99%	424,847.17	8.36%	
Hosta# (1,652,011.0 …	1,304	10	37.26%	27.25%	1,811,806.42	3.85%	
Triploid# (239,958.0 / …	88	1	2.51%	1.84%	284,344.39	10.07%	

Plot 2: D02 VL-MG-A Gated on (P1 in R1)	Count	Events / µL	% of This Plot	% of All	Mean FL2-A	CV FL2-A	Median FL2-A
This Plot	3,500	26	100.00%	73.15%	922,724.52	83.20%	

Hosta 'Praying Hands' genome size of 23.34 Gb (2n DNA content).

Sample calculation = 23.34 Gb Hosta 'Praying Hands' genome x (188,893.79/ 1,811,806.42) = 2.43 Gb (2n DNA content)

***Microchloa caffra* VL-MG-A genome size of 2.43 Gb (2C DNA content).**

D3 - VL-MG-B ☑ Show Statistics

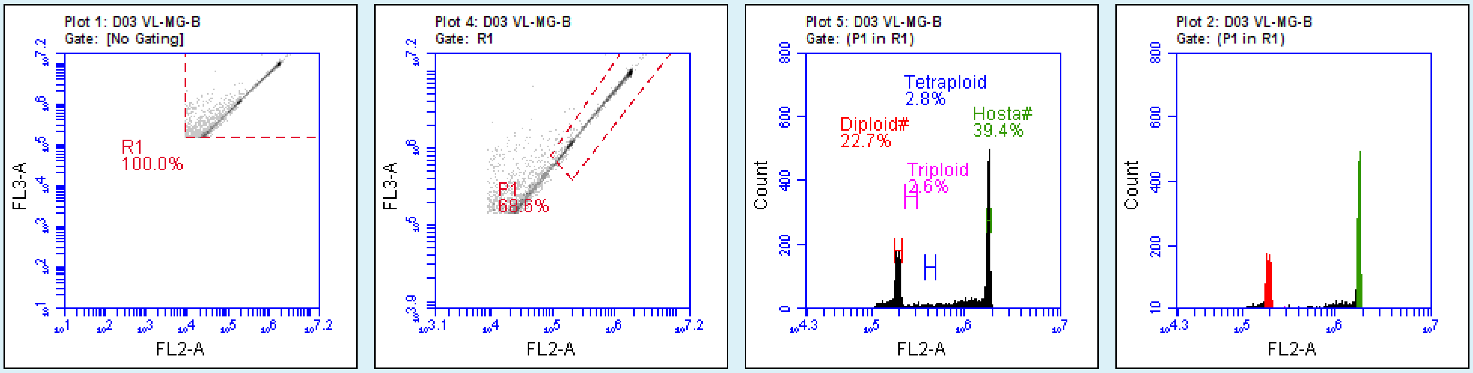

Plot 1: D03 VL-MG-B	Count	Events / µL	% of This Plot	% of All	Mean FL2-A	Mean FL3-A	CV FL2-A	CV FL3-A	Median FL2-A	Median FL3-A
All	5,104	41	100.00%	100.00%	743,863.71	3,958,390.56	105.54%	101.25%		
R1	5,104	41	100.00%	100.00%	743,863.71	3,958,390.56	105.54%	101.25%		

Plot 4: D03 VL-MG-B Gated on R1	Count	Events / µL	% of This Plot	% of All	Mean FL2-A	Mean FL3-A	CV FL2-A	CV FL3-A	Median FL2-A	Median FL3-A
This Plot	5,104	41	100.00%	100.00%	743,863.71	3,958,390.56	105.54%	101.25%		
P1	3,500	28	68.57%	68.57%	1,066,381.22	5,611,188.78	70.63%	68.12%		

Plot 5: D03 VL-MG-B Gated on (P1 in R1)	Count	Events / µL	% of This Plot	% of All	Mean FL2-A	CV FL2-A	Median FL2-A
This Plot	3,500	28	100.00%	68.57%	1,066,381.22	70.63%	
Diploid# (170,716.0 / …	796	6	22.74%	15.60%	190,008.38	5.23%	
Tetraploid (363,792.0 …	99	1	2.83%	1.94%	422,523.81	8.61%	
Hosta# (1,715,701.0 …	1,379	11	39.40%	27.02%	1,839,727.89	3.32%	
Triploid (222,473.0 / 3…	91	1	2.60%	1.78%	267,568.41	9.60%	

Plot 2: D03 VL-MG-B Gated on (P1 in R1)	Count	Events / µL	% of This Plot	% of All	Mean FL2-A	CV FL2-A	Median FL2-A
This Plot	3,500	28	100.00%	68.57%	1,066,381.22	70.63%	

Hosta ‘Praying Hands’ genome size of 23.34 Gb (2n DNA content).

Sample calculation = 23.34 Gb Hosta ‘Praying Hands’ genome x (190,008.38/ 1,839,727.89) = 2.41 Gb (2n DNA content)

***Microchloa caffra* VL-MG-B genome size of 2.41 Gb (2C DNA content).**

A3 - praying hands anbd tomato ☑ Show Statistics

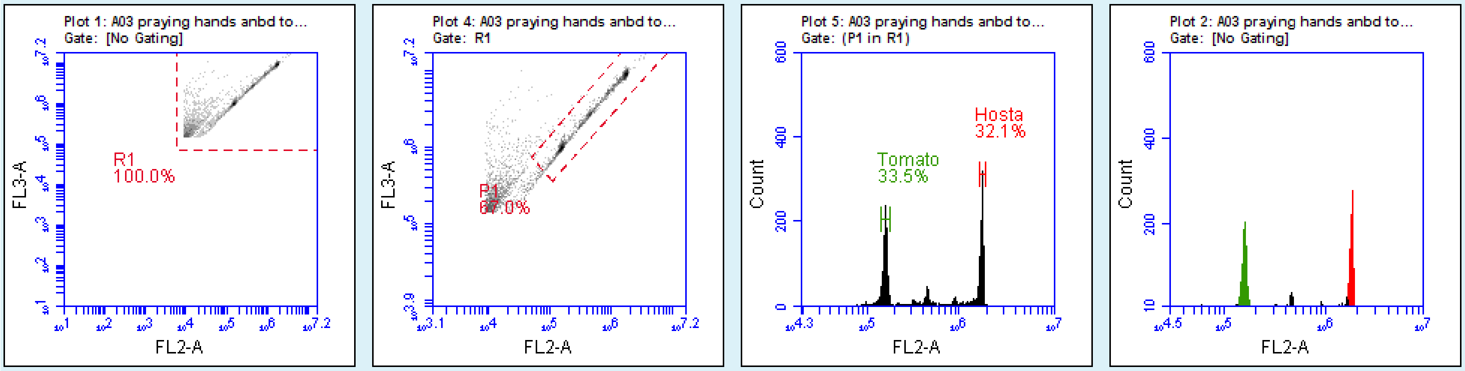

Plot 1: A03 praying hands anbd tomato	Count	Events / µL	% of This Plot	% of All	Mean FL2-A	Mean FL3-A	CV FL2-A	CV FL3-A	Median FL2-A	Median FL3-A
All	3,714	133	100.00%	100.00%	584,245.65	3,170,601.67	125.59%	113.66%		
R1	3,714	133	100.00%	100.00%	584,245.65	3,170,601.67	125.59%	113.66%		

Plot 4: A03 praying hands anbd tomato Gated on R1	Count	Events / µL	% of This Plot	% of All	Mean FL2-A	Mean FL3-A	CV FL2-A	CV FL3-A	Median FL2-A	Median FL3-A
This Plot	3,714	133	100.00%	100.00%	584,245.65	3,170,601.67	125.59%	113.66%		
P1	2,487	89	66.96%	66.96%	862,530.96	4,523,831.63	87.49%	81.66%		

Plot 5: A03 praying hands anbd tomato Gated on (P1 in R1)	Count	Events / µL	% of This Plot	% of All	Mean FL2-A	CV FL2-A	Median FL2-A
This Plot	2,487	89	100.00%	66.96%	862,530.96	87.49%	
Hosta (1,652,011.0 / 1,995,984.0)	799	29	32.13%	21.51%	1,791,359.02	3.54%	
Tomato (136,050.0 / 177,297.0)	832	30	33.45%	22.40%	155,023.40	5.26%	

Plot 2: A03 praying hands anbd tomato	Count	Events / µL	% of This Plot	% of All	Mean FL2-A	CV FL2-A	Median FL2-A
All	3,714	133	100.00%	100.00%	584,245.65	125.59%	

2.02 Gb Tomato seed genome, Green peak 2C DNA content

Sample calculation = 2.02Gb Tomato seed genome x (1,791,359.02/ 155,023.40) = 23.34 Gb (2n DNA content) or (11.67 Gb / haploid 1C DNA genome)

Hosta 'Praying Hands' genome size of 23.34 Gb (2n DNA content)

A5 - BK-DI-C ☑ Show Statistics

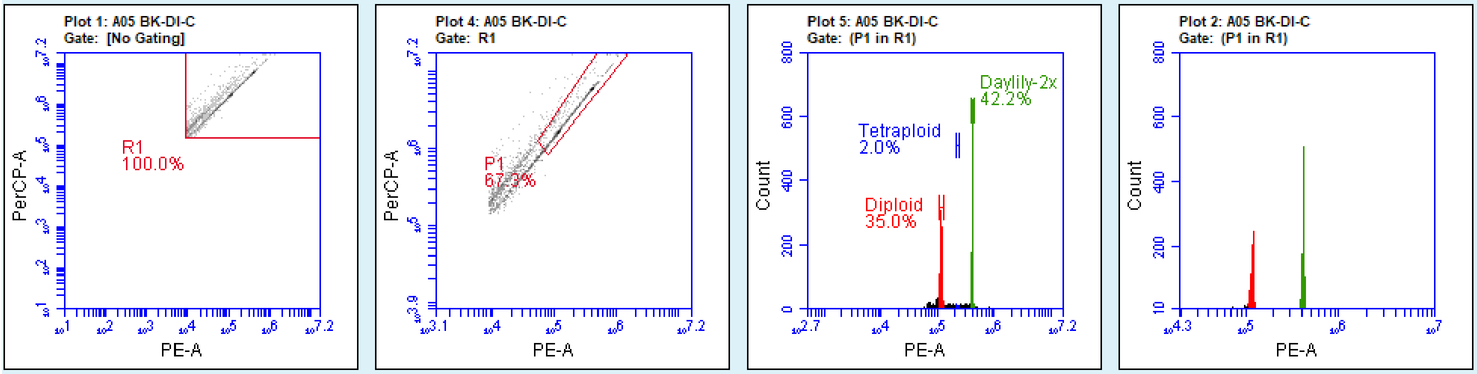

Plot 1: A05 BK-DI-C	Count	Events / µL	% of This Plot	% of All	Mean PE-A	Mean PerCP-A	CV PE-A	CV PerCP-A	Median PE-A	Median PerCP-A
All	3,011	86	100.00%	100.00%	186,988.39	2,792,088.56	92.11%	89.69%		
R1	3,011	86	100.00%	100.00%	186,988.39	2,792,088.56	92.11%	89.69%		

Plot 4: A05 BK-DI-C Gated on R1	Count	Events / µL	% of This Plot	% of All	Mean PE-A	Mean PerCP-A	CV PE-A	CV PerCP-A	Median PE-A	Median PerCP-A
This Plot	3,011	86	100.00%	100.00%	186,988.39	2,792,088.56	92.11%	89.69%		
P1	2,026	58	67.29%	67.29%	264,331.63	3,840,661.01	60.42%	60.64%		

Plot 5: A05 BK-DI-C Gated on (P1 in R1)	Count	Events / µL	% of This Plot	% of All	Mean PE-A	CV PE-A	Median PE-A
This Plot	2,026	58	100.00%	67.29%	264,331.63	60.42%	
Diploid (103,687.0 / …	709	20	35.00%	23.55%	115,554.62	4.45%	
Tetraploid (205,141.…	41	1	2.02%	1.36%	225,416.27	4.94%	
Daylily-2x (381,454.…	855	24	42.20%	28.40%	413,229.82	2.55%	

Plot 2: A05 BK-DI-C Gated on (P1 in R1)	Count	Events / µL	% of This Plot	% of All	Mean PE-A	CV PE-A	Median PE-A
This Plot	2,026	58	100.00%	67.29%	264,331.63	60.42%	

Daylily ‘Purple Pixie Gumdrop’ genome size of 8.59 Gb (2C DNA content).

Sample calculation = 8.59 Gb Daylily ‘Purple Pixie Gumdrop’ genome x (115,554.62/ 413,229.82) = 2.40 Gb (2n DNA content)

***Microchloa caffra* BK-DI-C genome size of 2.40 Gb (2C DNA content).**

A6 - BK-UK-C ☑ Show Statistics

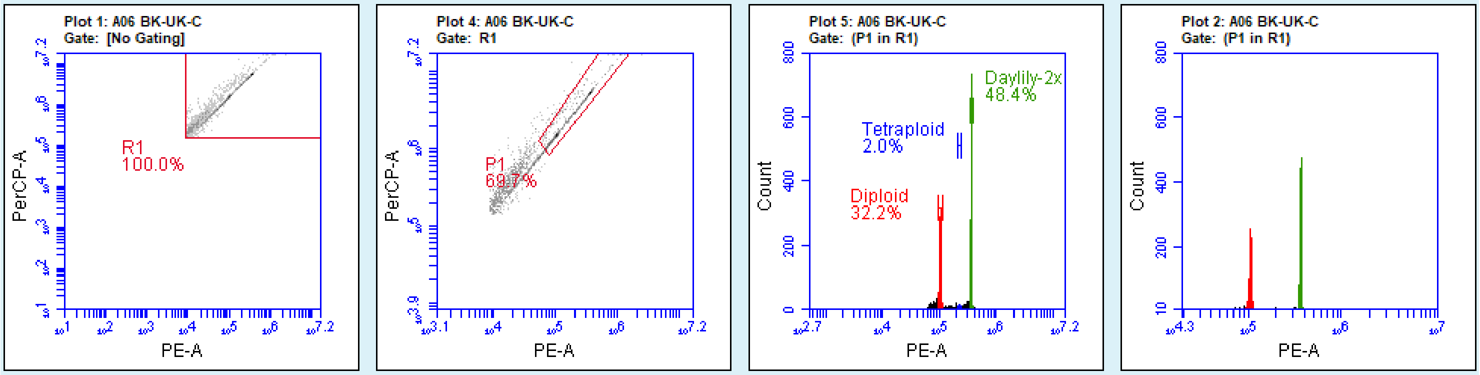

Plot 1: A06 BK-UK-C	Count	Events / µL	% of This Plot	% of All	Mean PE-A	Mean PerCP-A	CV PE-A	CV PerCP-A	Median PE-A	Median PerCP-A
All	3,073	70	100.00%	100.00%	182,156.37	2,710,443.93	88.40%	83.71%		
R1	3,073	70	100.00%	100.00%	182,156.37	2,710,443.93	88.40%	83.71%		

Plot 4: A06 BK-UK-C Gated on R1	Count	Events / µL	% of This Plot	% of All	Mean PE-A	Mean PerCP-A	CV PE-A	CV PerCP-A	Median PE-A	Median PerCP-A
This Plot	3,073	70	100.00%	100.00%	182,156.37	2,710,443.93	88.40%	83.71%		
P1	2,141	49	69.67%	69.67%	248,783.99	3,636,851.79	55.98%	56.31%		

Plot 5: A06 BK-UK-C Gated on (P1 in R1)	Count	Events / µL	% of This Plot	% of All	Mean PE-A	CV PE-A	Median PE-A
This Plot	2,141	49	100.00%	69.67%	248,783.99	55.98%	
Diploid (91,589.0 / 11...	690	16	32.23%	22.45%	103,207.27	3.80%	
Tetraploid (205,141....	42	1	1.96%	1.37%	225,816.10	4.85%	
Daylily-2x (336,949.0...	1,037	24	48.44%	33.75%	362,502.55	2.26%	

Plot 2: A06 BK-UK-C Gated on (P1 in R1)	Count	Events / µL	% of This Plot	% of All	Mean PE-A	CV PE-A	Median PE-A
This Plot	2,141	49	100.00%	69.67%	248,783.99	55.98%	

Daylily 'Purple Pixie Gumdrop' genome size of 8.59 Gb (2C DNA content).

Sample calculation = 8.59 Gb Daylily 'Purple Pixie Gumdrop' genome x (103,207.27 / 362,502.55) = 2.46 Gb (2n DNA content)

Microchloa caffra **BK-UK-C genome size of 2.46 Gb (2C DNA content).**

B1 - Mtn-Sis-C ☑ Show Statistics

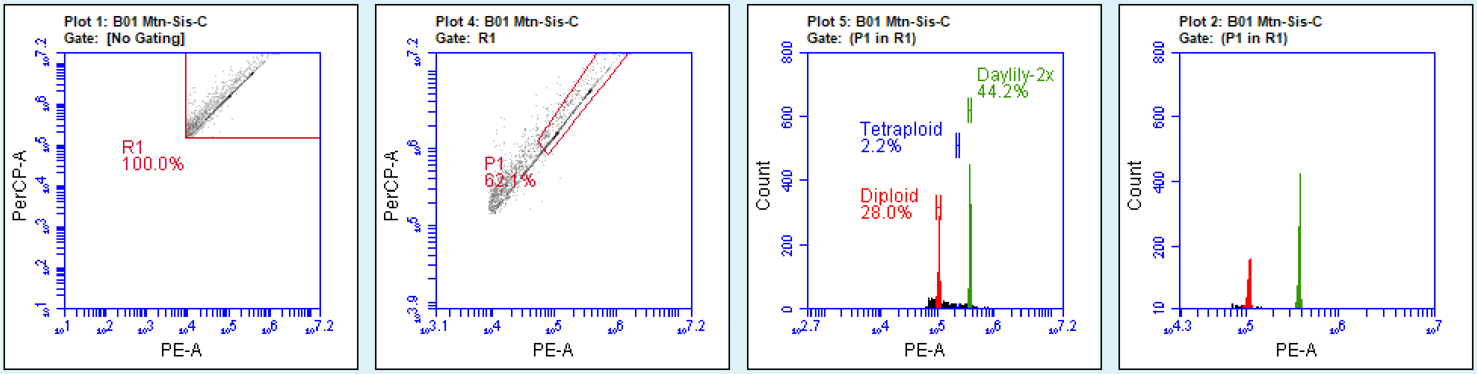

Plot 1: B01 Mtn-Sis-C	Count	Events / µL	% of This Plot	% of All	Mean PE-A	Mean PerCP-A	CV PE-A	CV PerCP-A	Median PE-A	Median PerCP-A
All	3,098	163	100.00%	100.00%	171,720.52	2,641,474.69	100.56%	95.45%		
R1	3,098	163	100.00%	100.00%	171,720.52	2,641,474.69	100.56%	95.45%		

Plot 4: B01 Mtn-Sis-C Gated on R1	Count	Events / µL	% of This Plot	% of All	Mean PE-A	Mean PerCP-A	CV PE-A	CV PerCP-A	Median PE-A	Median PerCP-A
This Plot	3,098	163	100.00%	100.00%	171,720.52	2,641,474.69	100.56%	95.45%		
P1	1,924	101	62.10%	62.10%	256,772.78	3,836,199.89	60.64%	61.53%		

Plot 5: B01 Mtn-Sis-C Gated on (P1 in R1)	Count	Events / µL	% of This Plot	% of All	Mean PE-A	CV PE-A	Median PE-A
This Plot	1,924	101	100.00%	62.10%	256,772.78	60.64%	
Diploid (91,589.0 / 117...	538	28	27.96%	17.37%	105,732.29	4.83%	
Tetraploid (205,141.0 /...	42	2	2.18%	1.36%	225,520.07	5.86%	
Daylily-2x (336,949.0 /...	851	45	44.23%	27.47%	376,909.92	3.09%	

Plot 2: B01 Mtn-Sis-C Gated on (P1 in R1)	Count	Events / µL	% of This Plot	% of All	Mean PE-A	CV PE-A	Median PE-A
This Plot	1,924	101	100.00%	62.10%	256,772.78	60.64%	

Daylily ‘Purple Pixie Gumdrop’ genome size of 8.59 Gb (2C DNA content).

Sample calculation = 8.59 Gb Daylily ‘Purple Pixie Gumdrop’ genome x (105,732.29 / 376,909.92) = 2.41 Gb (2n DNA content)

***Microchloa caffra* Mtn-Sis-C genome size of 2.41 Gb (2C DNA content).**

B2 - SS-LT-B ☑ Show Statistics

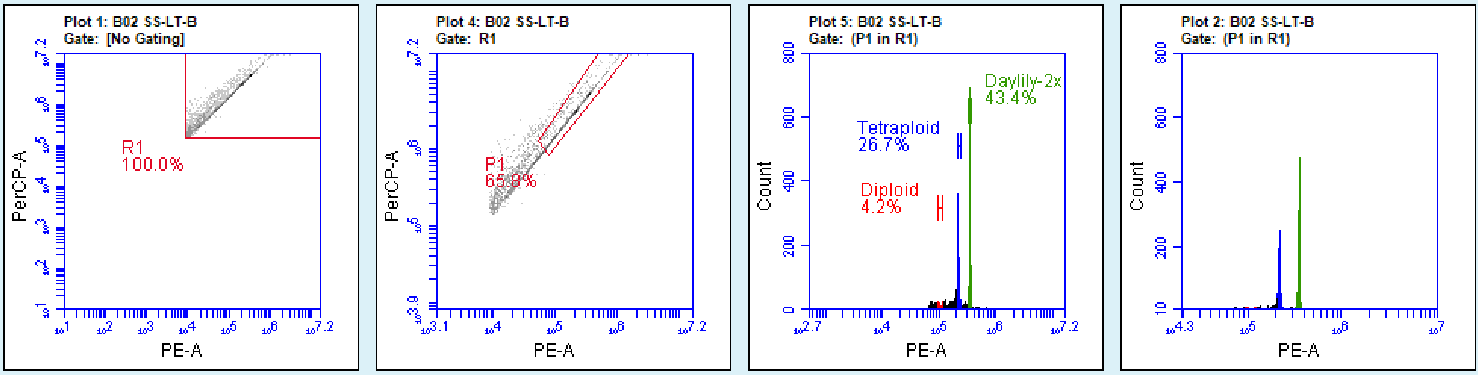

Plot 1: B02 SS-LT-B	Count	Events / µL	% of This Plot	% of All	Mean PE-A	Mean PerCP-A	CV PE-A	CV PerCP-A	Median PE-A	Median PerCP-A
All	3,183	138	100.00%	100.00%	191,381.60	2,876,830.65	89.31%	78.93%		
R1	3,183	138	100.00%	100.00%	191,381.60	2,876,830.65	89.31%	78.93%		

Plot 4: B02 SS-LT-B Gated on R1	Count	Events / µL	% of This Plot	% of All	Mean PE-A	Mean PerCP-A	CV PE-A	CV PerCP-A	Median PE-A	Median PerCP-A
This Plot	3,183	138	100.00%	100.00%	191,381.60	2,876,830.65	89.31%	78.93%		
P1	2,095	91	65.82%	65.82%	270,188.32	3,982,169.52	44.99%	46.37%		

Plot 5: B02 SS-LT-B Gated on (P1 in R1)	Count	Events / µL	% of This Plot	% of All	Mean PE-A	CV PE-A	Median PE-A
This Plot	2,095	91	100.00%	65.82%	270,188.32	44.99%	
Diploid (91,589.0 / 1…	89	4	4.25%	2.80%	103,602.29	8.18%	
Tetraploid (205,141.…	560	24	26.73%	17.59%	216,985.84	3.55%	
Daylily-2x (316,683.…	910	40	43.44%	28.59%	350,445.85	2.57%	

Plot 2: B02 SS-LT-B Gated on (P1 in R1)	Count	Events / µL	% of This Plot	% of All	Mean PE-A	CV PE-A	Median PE-A
This Plot	2,095	91	100.00%	65.82%	270,188.32	44.99%	

Daylily ‘Purple Pixie Gumdrop’ genome size of 8.59 Gb (2C DNA content).

Sample calculation = 8.59 Gb Daylily ‘Purple Pixie Gumdrop’ genome x (216,985.84 / 350,445.85) = 5.32 Gb (2n DNA content)

***Microchloa caffra* SS-LT-B genome size of 5.32 Gb (2C DNA content).**

B3 - SS-RV-C ☑ Show Statistics

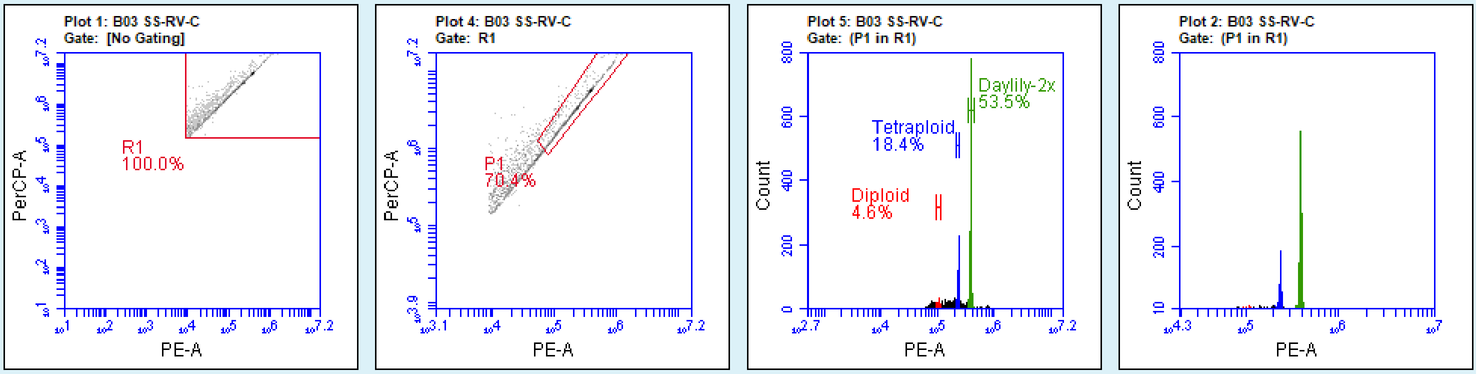

Plot 1: B03 SS-RV-C	Count	Events / µL	% of This Plot	% of All	Mean PE-A	Mean PerCP-A	CV PE-A	CV PerCP-A	Median PE-A	Median PerCP-A
All	2,973	83	100.00%	100.00%	227,291.86	3,359,903.80	75.23%	72.49%		
R1	2,973	83	100.00%	100.00%	227,291.86	3,359,903.80	75.23%	72.49%		

Plot 4: B03 SS-RV-C Gated on R1	Count	Events / µL	% of This Plot	% of All	Mean PE-A	Mean PerCP-A	CV PE-A	CV PerCP-A	Median PE-A	Median PerCP-A
This Plot	2,973	83	100.00%	100.00%	227,291.86	3,359,903.80	75.23%	72.49%		
P1	2,093	58	70.40%	70.40%	310,147.22	4,500,854.82	43.45%	43.72%		

Plot 5: B03 SS-RV-C Gated on (P1 in R1)	Count	Events / µL	% of This Plot	% of All	Mean PE-A	CV PE-A	Median PE-A
This Plot	2,093	58	100.00%	70.40%	310,147.22	43.45%	
Diploid (91,589.0 / 11…	97	3	4.63%	3.26%	104,003.69	6.71%	
Tetraploid (205,141.…	386	11	18.44%	12.98%	232,608.01	3.70%	
Daylily-2x (336,949.0…	1,120	31	53.51%	37.67%	387,504.56	3.41%	

Plot 2: B03 SS-RV-C Gated on (P1 in R1)	Count	Events / µL	% of This Plot	% of All	Mean PE-A	CV PE-A	Median PE-A
This Plot	2,093	58	100.00%	70.40%	310,147.22	43.45%	

Daylily ‘Purple Pixie Gumdrop’ genome size of 8.59 Gb (2C DNA content).

Sample calculation = 8.59 Gb Daylily ‘Purple Pixie Gumdrop’ genome x (232,608.01 / 387,504.56) = 5.16 Gb (2n DNA content)

***Microchloa caffra* SS-RV-C genome size of 5.16 Gb (2C DNA content).**

B4 - VL Falls-B ☑ Show Statistics

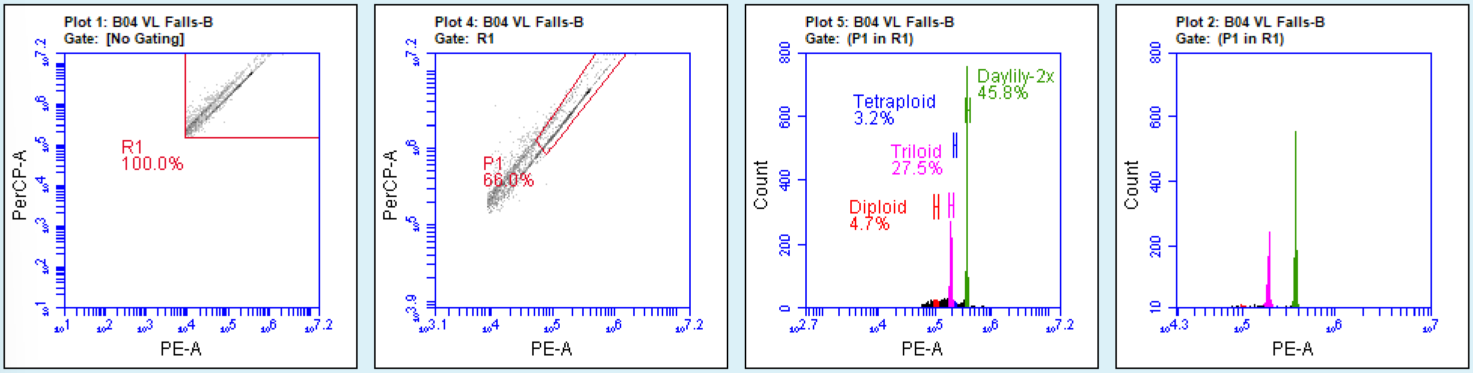

Plot 1: B04 VL Falls-B	Count	Events / µL	% of This Plot	% of All	Mean PE-A	Mean PerCP-A	CV PE-A	CV PerCP-A	Median PE-A	Median PerCP-A
All	3,248	64	100.00%	100.00%	191,236.54	2,902,133.97	83.27%	79.39%		
R1	3,248	64	100.00%	100.00%	191,236.54	2,902,133.97	83.27%	79.39%		

Plot 4: B04 VL Falls-B Gated on R1	Count	Events / µL	% of This Plot	% of All	Mean PE-A	Mean PerCP-A	CV PE-A	CV PerCP-A	Median PE-A	Median PerCP-A
This Plot	3,248	64	100.00%	100.00%	191,236.54	2,902,133.97	83.27%	79.39%		
P1	2,143	42	65.98%	65.98%	274,543.78	4,075,533.35	45.33%	47.32%		

Plot 5: B04 VL Falls-B Gated on (P1 in R1)	Count	Events / µL	% of This Plot	% of All	Mean PE-A	CV PE-A	Median PE-A
This Plot	2,143	42	100.00%	65.98%	274,543.78	45.33%	
Diploid (91,589.0 / 117…	100	2	4.67%	3.08%	103,478.34	6.98%	
Tetraploid (205,141.0 /…	68	1	3.17%	2.09%	222,628.13	5.16%	
Daylily-2x (336,949.0 /…	982	19	45.82%	30.23%	371,158.14	2.78%	
Triloid (170,308.0 / 218…	590	12	27.53%	18.17%	190,966.47	4.16%	

Plot 2: B04 VL Falls-B Gated on (P1 in R1)	Count	Events / µL	% of This Plot	% of All	Mean PE-A	CV PE-A	Median PE-A
This Plot	2,143	42	100.00%	65.98%	274,543.78	45.33%	

Daylily ‘Purple Pixie Gumdrop’ genome size of 8.59 Gb (2C DNA content).

Sample calculation = 8.59 Gb Daylily ‘Purple Pixie Gumdrop’ genome x (190,966.47 / 371,158.14) = 4.41 Gb (2n DNA content)

Microchloa caffra **VL Falls-B genome size of 4.41 Gb (2C DNA content).**

B5 - VL MG-C ☑ Show Statistics

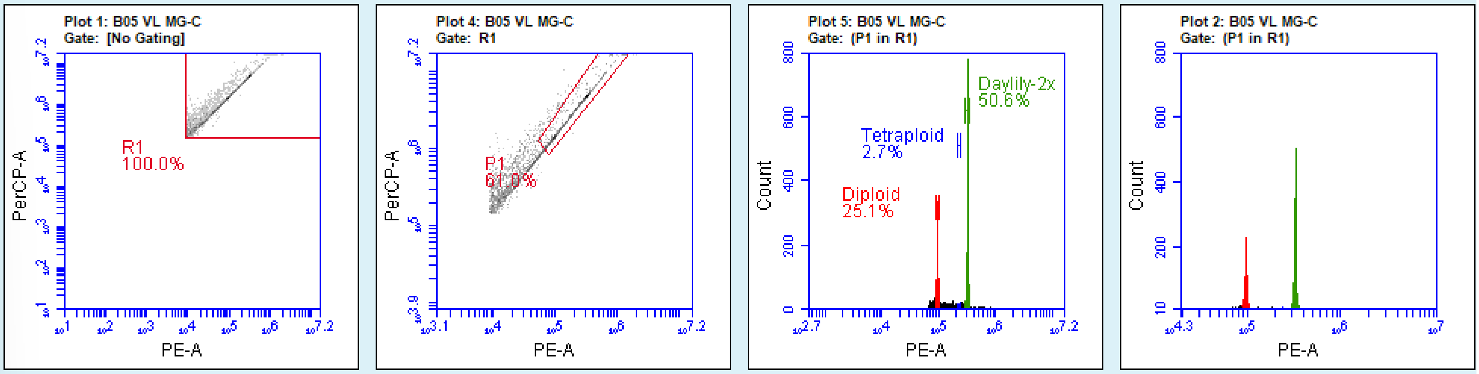

Plot 1: B05 VL MG-C	Count	Events / µL	% of This Plot	% of All	Mean PE-A	Mean PerCP-A	CV PE-A	CV PerCP-A	Median PE-A	Median PerCP-A
All	3,465	99	100.00%	100.00%	160,632.89	2,458,817.93	100.24%	94.89%		
R1	3,465	99	100.00%	100.00%	160,632.89	2,458,817.93	100.24%	94.89%		

Plot 4: B05 VL MG-C Gated on R1	Count	Events / µL	% of This Plot	% of All	Mean PE-A	Mean PerCP-A	CV PE-A	CV PerCP-A	Median PE-A	Median PerCP-A
This Plot	3,465	99	100.00%	100.00%	160,632.89	2,458,817.93	100.24%	94.89%		
P1	2,113	60	60.98%	60.98%	243,923.51	3,622,200.09	59.07%	60.51%		

Plot 5: B05 VL MG-C Gated on (P1 in R1)	Count	Events / µL	% of This Plot	% of All	Mean PE-A	CV PE-A	Median PE-A
This Plot	2,113	60	100.00%	60.98%	243,923.51	59.07%	
Diploid (86,081.0 / 10…	530	15	25.08%	15.30%	94,801.61	3.58%	
Tetraploid (205,141.0…	57	2	2.70%	1.65%	226,698.56	5.19%	
Daylily-2x (279,735.0 …	1,070	31	50.64%	30.88%	324,406.36	3.30%	
NullSubset							

Plot 2: B05 VL MG-C Gated on (P1 in R1)	Count	Events / µL	% of This Plot	% of All	Mean PE-A	CV PE-A	Median PE-A
This Plot	2,113	60	100.00%	60.98%	243,923.51	59.07%	

Daylily 'Purple Pixie Gumdrop' genome size of 8.59 Gb (2C DNA content).

Sample calculation = 8.59 Gb Daylily 'Purple Pixie Gumdrop' genome x (94,801.61 / 324,406.36) = 2.51 Gb (2n DNA content)

Microchloa caffra **VL MG-C genome size of 2.51 Gb (2C DNA content).**

If needed, convert DNA mass in picograms to the numbers of base pairs or vice versa as follows 1 pg DNA = .978 x 10^9 Bp.

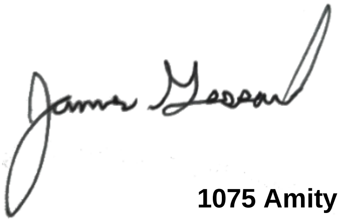

James Gossard, Laboratory Director

1075 Amity Road * Galloway, Ohio 43119 * (614)419-1781 * (614)851-1375 Fax

Additional standard

A4 - daylilyppgandtomato ☑ Show Statistics

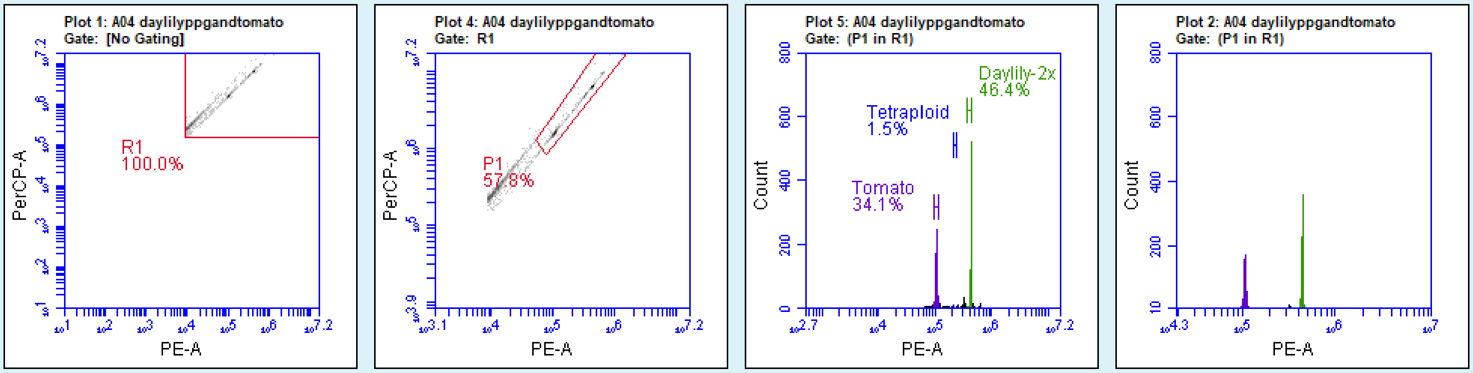

Plot 1: A04 daylilyppgandtomato	Count	Events / µL	% of This Plot	% of All	Mean PE-A	Mean PerCP-A	CV PE-A	CV PerCP-A	Median PE-A	Median PerCP-A
All	2,459	91	100.00%	100.00%	177,499.91	2,640,742.93	107.25%	101.92%		
R1	2,459	91	100.00%	100.00%	177,499.91	2,640,742.93	107.25%	101.92%		

Plot 4: A04 daylilyppgandtomato Gated on R1	Count	Events / µL	% of This Plot	% of All	Mean PE-A	Mean PerCP-A	CV PE-A	CV PerCP-A	Median PE-A	Median PerCP-A
This Plot	2,459	91	100.00%	100.00%	177,499.91	2,640,742.93	107.25%	101.92%		
P1	1,421	53	57.79%	57.79%	295,390.36	4,297,461.99	58.36%	56.99%		

Plot 5: A04 daylilyppgandtomato Gated on (P1 in R1)	Count	Events / µL	% of This Plot	% of All	Mean PE-A	CV PE-A	Median PE-A
This Plot	1,421	53	100.00%	57.79%	295,390.36	58.36%	
Tomato (91,589.0 / 117,382.0)	485	18	34.13%	19.72%	104,748.71	3.97%	
Tetraploid (205,141.0 / 247,098.0)	22	1	1.55%	0.89%	220,431.23	5.48%	
Daylily-2x (358,511.0 / 459,472.0)	660	24	46.45%	26.84%	438,807.89	2.75%	

Plot 2: A04 daylilyppgandtomato Gated on (P1 in R1)	Count	Events / µL	% of This Plot	% of All	Mean PE-A	CV PE-A	Median PE-A
This Plot	1,421	53	100.00%	57.79%	295,390.36	58.36%	

Daylily ‘Purple Pixie Gumdrop’ genome size of 8.59 Gb (2C DNA content).

Sample calculation = 2.05 Gb Tomato seed genome x (438,807.89/104,748.71) = 8.59 Gb (2C DNA content)

A3 - tomato ☑ Show Statistics

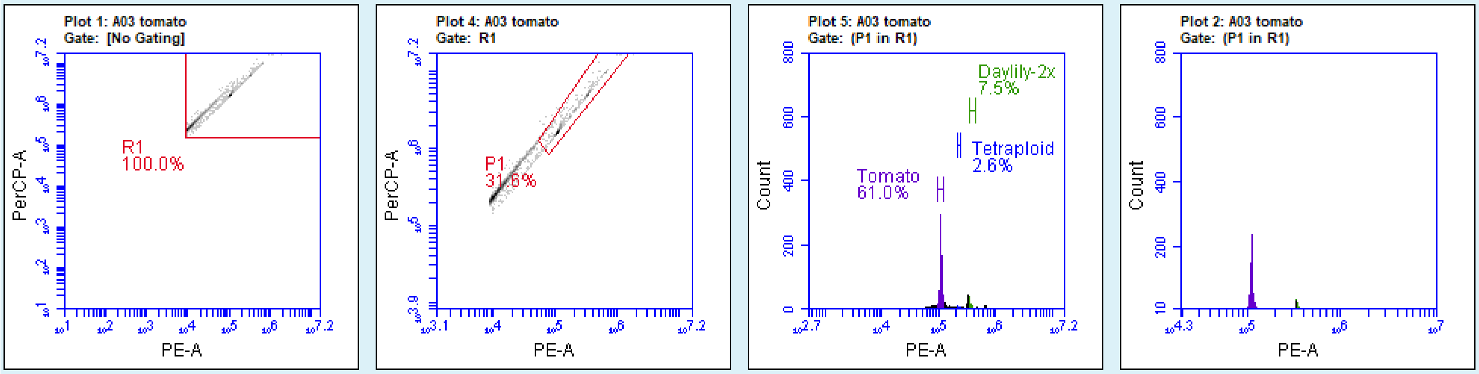

Plot 1: A03 tomato	Count	Events / µL	% of This Plot	% of All	Mean PE-A	Mean PerCP-A	CV PE-A	CV PerCP-A	Median PE-A	Median PerCP-A
All	3,039	95	100.00%	100.00%	66,516.46	1,113,979.21	167.97%	145.79%		
R1	3,039	95	100.00%	100.00%	66,516.46	1,113,979.21	167.97%	145.79%		

Plot 4: A03 tomato Gated on R1	Count	Events / µL	% of This Plot	% of All	Mean PE-A	Mean PerCP-A	CV PE-A	CV PerCP-A	Median PE-A	Median PerCP-A
This Plot	3,039	95	100.00%	100.00%	66,516.46	1,113,979.21	167.97%	145.79%		
P1	959	30	31.56%	31.56%	172,632.43	2,663,712.38	80.13%	78.91%		

Plot 5: A03 tomato Gated on (P1 in R1)	Count	Events / µL	% of This Plot	% of All	Mean PE-A	CV PE-A	Median PE-A
This Plot	959	30	100.00%	31.56%	172,632.43	80.13%	
Tomato (91,589.0 / …	585	18	61.00%	19.25%	109,108.77	4.81%	
Tetraploid (205,141.…	25	1	2.61%	0.82%	220,975.96	5.00%	
Daylily-2x (336,949.…	72	2	7.51%	2.37%	363,570.51	7.81%	

Plot 2: A03 tomato Gated on (P1 in R1)	Count	Events / µL	% of This Plot	% of All	Mean PE-A	CV PE-A	Median PE-A
This Plot	959	30	100.00%	31.56%	172,632.43	80.13%	

Tomato seed (lot#: D21OH101NC) with an estimated genome size of 2.05 Gb (2n DNA content) (Michaelson et al., 1991; Sato et al., 2012) was included as an internal control for each sample for genome size.

Standard used by The Ohio State University

Polyploidy induced by colchicine in Taraxacum kok-saghyz and its effects on morphological and biochemical traits Zinan Luo, Brian J. Iaffaldano, Katrina Cornish*

Method

Read this package insert carefully before use.

CyStain™ PI Absolute P REF 05-5022

INTENDED PURPOSE

For Research Use Only. Not for use in diagnostic procedures.

CyStain™ PI Absolute P is a reagent kit for nuclei extraction and DNA staining of nuclear DNA from different plant species and tissues in order to determine absolute or relative genome size and ploidy level. It is a staining protocol for the fluorescent staining of nuclear DNA of fixed and non-fixed cells from different origin. The kit was developed for the use in cells obtained from cell suspensions and solid tissues, respectively. The procedure may include fixation with 70 % ethanol. Prepared samples can be analysed on standard flow cytometers.

Intended to be used by professionals or trained personnel only.

KIT COMPONENTS

Packing contains reagents for 250 tests:

- 125 ml Nuclei Extraction Buffer
- 500 ml Staining Buffer
- 2 x 1.5 ml Propidium Iodide
- 1 x 5 mg RNase A

PRINCIPLE OF EXAMINATION METHOD

Reagent kit for nuclei extraction and DNA staining of nuclear DNA from plant tissues to determine absolute and relative genome size and ploidy level.

ADDITIONAL REQUIRED EQUIPMENT

- A calibrated pipette and pipette tips
- Sample tube(s) compliant to the flow cytometer
- Personal protective equipment

INSTRUMENT REQUIREMENTS

A flow cytometer with 488 nm or 532 nm laser light source and a fluorescence parameter of orange - red fluorescence emission (> 590 nm).

INSTRUCTIONS

For instrument alignment and quality control, please refer to the IFU of your flow cytometer.

sysmex

Preparation of RNase A Stock Solution

1. Add 1.5 ml H_2O to the tube containing RNase A).
2. Mix thoroughly.

Store RNase A stock solution at -20 °C.

Preparation of Staining Solution

The following preparation will suffice for 10 samples.

1. Add 120 µl Propidium Iodide and 60 µl RNase A Stock Solution to 20 ml Staining Buffer.

Freshly prepared Staining Solution is stable for 24 hours, if stored at 2-8 °C and protected from light.

Preparation and staining of samples

1. Put approximately 0.5 cm^2 (or less) of leaf tissue or other plant material in a plastic petri dish (REF No. 04-2005).
2. Add 3 to 4 drops of 500 µl Nuclei Extraction Buffer onto the plant material.
3. Chop the sample by using a sharp razor blade for 30-60 seconds (Razor blades need to be exchanged after 5-10 samples).
4. Add the remaining Nuclei Extraction Buffer and incubate for 30-90 seconds.
5. Filter sample through 50 µm CellTrics™ filter (REF No. 04-0042-2317) into a Sample tube (REF No. 04-2000).
6. Add 2 ml Staining Solution.
7. Incubate for 30-60 minutes protected from light at room temperature.
8. Start analysis.

If samples do show oxidation or staining inhibition effects, e.g. samples showing brownish color, instable peak positions or peaks with a significant left shoulder we do recommend to use as alternative reagent CyStain™ PI OxProtect (REF No. 05-5027).

STORAGE AND STABILITY

Storage

2-8 °C in the dark

Shelf life

Please refer to the expiry date, labelled on the bottle(s).

Reagent solutions

RNase A stock solution has to be stored at -20 °C.

Prepared Staining Solution has to be stored at 2-8 °C in the dark and is stable for 24 hours only.

**Laser excitation**	488 nm<br>640 nm
**Laser profile**	Blue laser beam: 9 x 94 µm<br>Red laser beam: 11 x 104 µm
**Laser power**	488 nm solid-state blue laser: 20 mW<br>640 nm diode red laser: 12.5 mW

Item	Specification
**Emission detection**	4 colors, standard optical filters<br>• FL1 533/30 nm<br>• FL2 585/40 nm<br>• FL3 >670 nm<br>• FL4 675/25 nm
**Optical alignment**	Fixed alignment
**Flow cell**	200-µm ID quartz capillary
**Minimum detectable particle size**	0.5 µm
**Minimum sample volume**	50 µL (12 x 75-mm tubes)<br>150 µL (BD Trucount tubes)<br>100 µL (CSampler Plus, 12 x 75-mm tubes)
**Flow rate**	14–66 µL/min, depending on the test
**Maximum events/sample**	1 million events
**Fluorescence sensitivity MESF***	FITC <75<br>PE <50
**Fluorescence linearity**	2 ±0.05% for chicken erythrocyte nuclei (CEN)
**Fluorescence precision**	≤3% CV for CEN
**Data acquisition rate**	10,000 events/s, maximum
**Fluid bottle capacity**	2 L sheath, 2 L waste<br>250 mL BD FACSClean<br>250 mL Detergent Solution
