# Supplementary material for: Higher order polyploids exhibit enhanced desiccation tolerance in the grass Microchloa caffra
Source: J Exp Bot. 2024 Mar 21;75(11):3612–23. doi: 10.1093/jxb/erae126 (PMC11156804; doi:10.1093/jxb/erae126)
Supplement: erae126_suppl_Supplementary_Figure_S1-S3_Table_S1 [file erae126_suppl_supplementary_figure_s1-s3_table_s1.pdf]

SUPPLEMENTARY INFORMATION

Higher order polyploids exhibit enhanced desiccation tolerance in the grass *Microchloa caffra*

Rose A. Marks[1,2,3], Paula Delgado[3], Givemore Munashe Makonya[3,4], Keren Cooper[3], Robert VanBuren[1,2], and Jill M. Farrant[3]

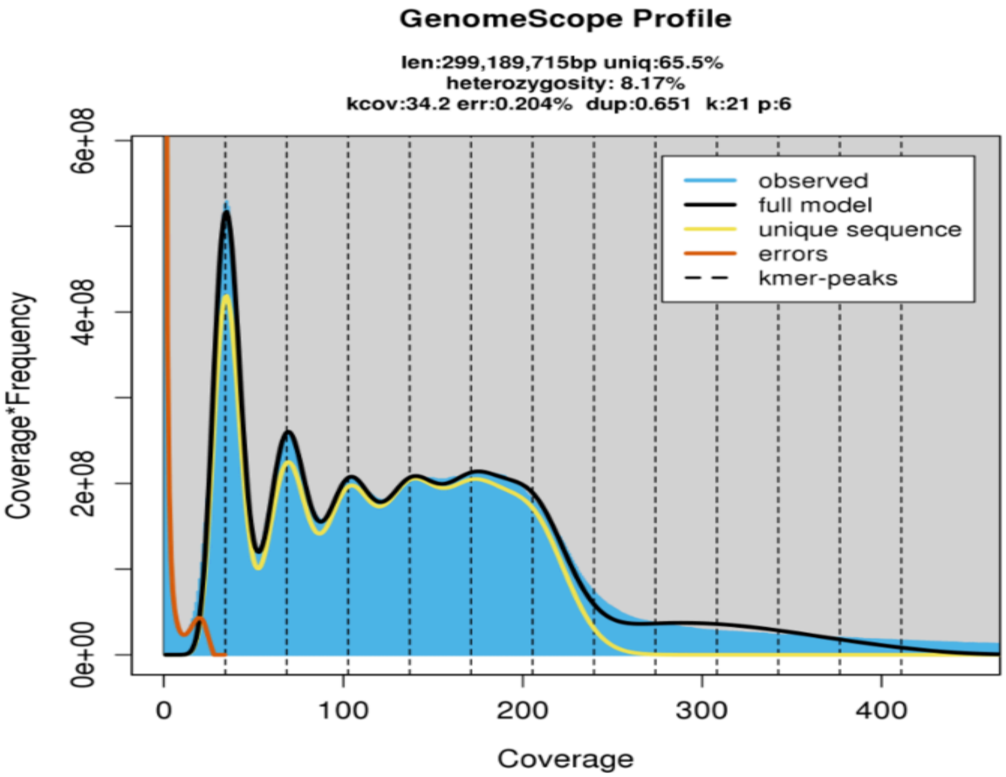

Figure S1. K-mer analyses of *M. caffra* whole genome sequencing data. A histogram of the observed K-mer distribution (blue) is plotted for high coverage HiFi whole genome sequencing data. The distribution of low coverage K-mers corresponding to errors in the raw reads are shown in orange with unique sequences shown in yellow and the full model in black. K-mer peaks are shown by dotted ones and the six peaks indicate that *M. caffra* is a hexaploid (2n=6) with a monoploid genome size of ~300-400 Mb. The prevalence of ‘aaab’ over ‘aabb’ alleles is consistent with autopolyploidy.

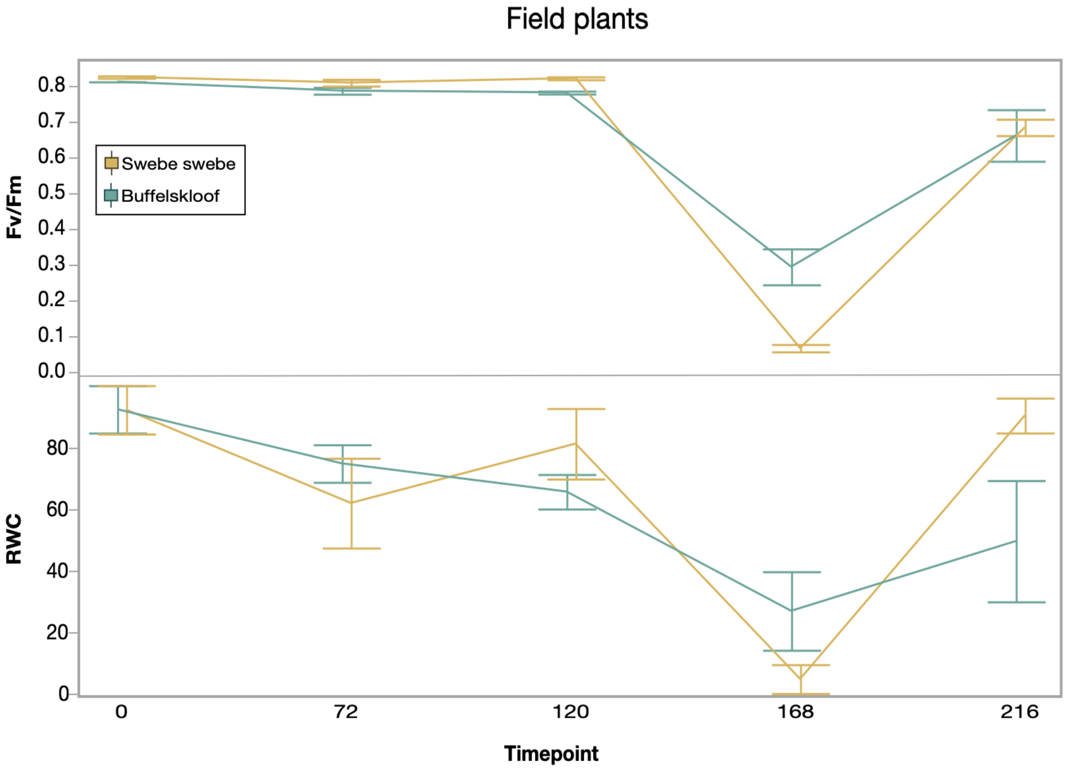

Figure S2. Physiological characterization of drying timecourse. Changes in photochemical efficiency of PSII (F_v/F_m) and relative water content (RWC) of field collected plants during a desiccation treatment imposed at the University of Cape Town. Selected plants from two sites (5 from Buffelskloof, Mpumalanga and 6 from Swebe Swebe, Limpopo) were desiccated under controlled conditions and rehydrated after 168 hours. Error bars represent standard error of the mean.

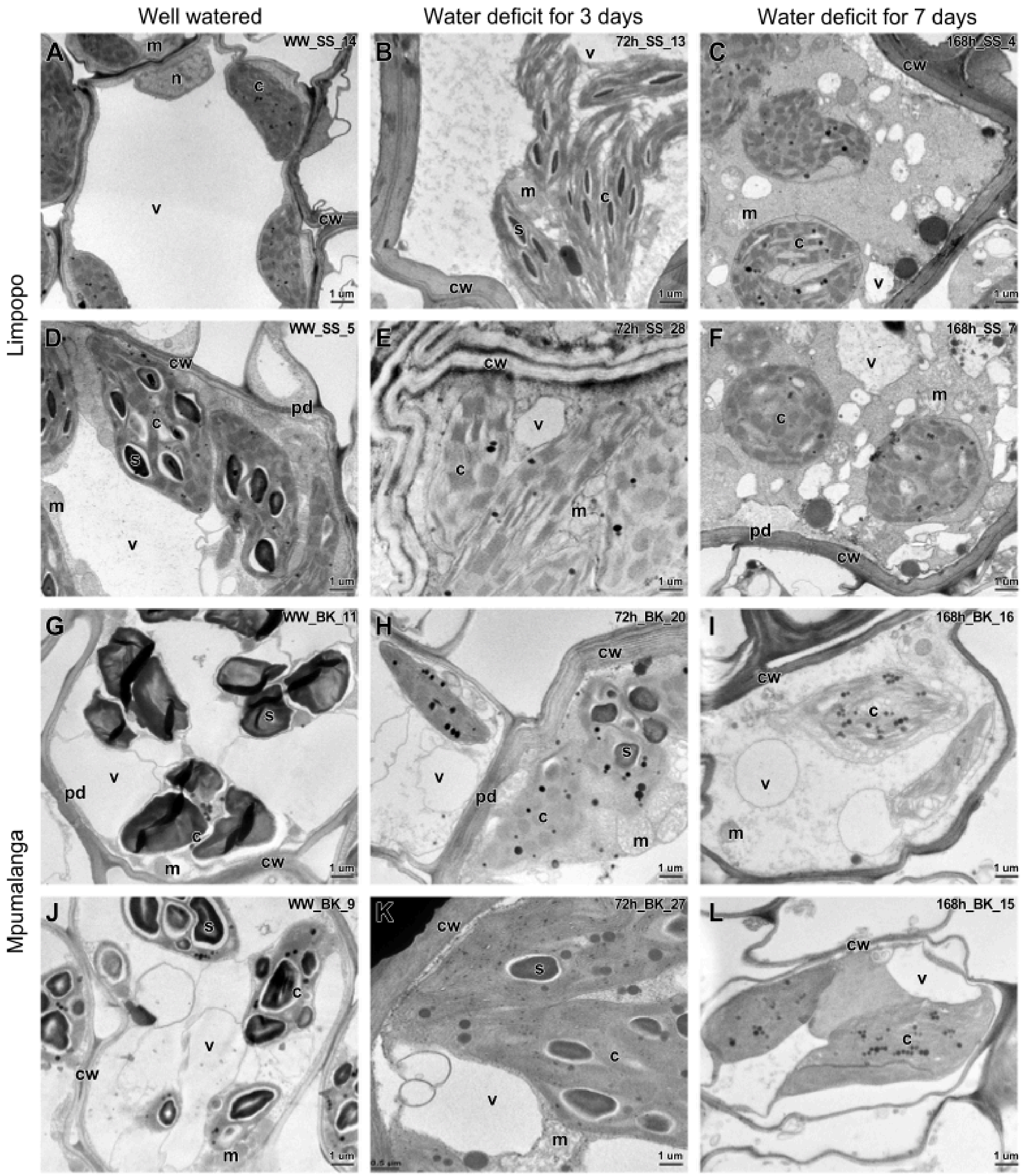

Figure S3. Transmission electron micrographs of mesophyll cells of plants collected from Swebe Swebe, Limpopo (A-F) and Buffelskloof, Mpumalanga (G-L) under well watered conditions (A, D, G, J) partial water deficit (B, E, H, K), and substantial water deficit (C, F, I, L). Abbreviations: CW=cell wall; C=chloroplast; M=mitochondrion; PD=plasmodesmata; S=starch; V=vacuole.

Table S1: Collection locations, mean annual precipitation and temperature, geo coordinates, and elevation. Precipitation and temperature data were taken from worldclim (Fick and Hijmans, 2017). .
SS=Swebe Swebe, VL=Veloren,, and BK=Buffelskloof.

**Population name**	**Annual precip (mm)**	**Annual temp (C)**	**GPS Coordinates**	**Elevation (M)**
BK breakfast rock	924	17	25.19.785 S, 030.29.650 E	1304
BK drop in	939	15	25.18.041 S, 030.30.463 E	1515
BK low kloof	904	17	25.20.085 S, 030.29.385 E	1193
BK upper kloof	939	15	25.17.078 S, 030.30.579 E	1631
Mountain sister	645	18.5	24.29.520 S, 027.44.377 E	1498
SS brown river	513	19.7	23.45.032 S, 028.05.557 E	911
SS lazy tree	627	17.8	23.48.731 S, 028.04.277 E	1085
SS river view	580	18.4	23.47.418 S, 028.04.553 E	1083
SS wasp corner	599	18.2	23.51.36 S, 028.02.188 E	1278
VL falls	613	19	24.48.235 S, 028.22.622 E	1295
VL main gate	623	18.7	24.47.624 S, 028.21.328 E	1213
